# Evolution of an Apomixis-Specific Allele Class in Supernumerary Chromatin of Apomictic *Boechera*

**DOI:** 10.3389/fpls.2022.890038

**Published:** 2022-06-01

**Authors:** Martin Mau, Terezie M. Mandáková, Xingliang Ma, Jana Ebersbach, Lifang Zou, Martin A. Lysak, Timothy F. Sharbel

**Affiliations:** ^1^Apomixis Research Group, Leibniz Institute of Plant Genetics and Crop Plant Research (IPK), Gatersleben, Germany; ^2^Department of Plant Sciences, College of Agriculture and Bioresources, University of Saskatchewan, Saskatoon, SK, Canada; ^3^Central European Institute of Technology, Masaryk University, Brno, Czechia; ^4^Saskatoon Research and Development Centre, Saskatoon, SK, Canada

**Keywords:** apomixis, pollen, *UPGRADE2*, tapetum, heterochromatic chromosome, gene evolution, *Boechera*, supernumerary DNA

## Abstract

Asexual reproduction through seeds in plants (i.e., apomixis) is a heritable trait, and apomixis- linked loci have been identified in multiple species. However, direct identification of genomic elements is typically hindered as apomixis-linked loci and are commonly found in recombination-suppressed and repetitive regions. Heterochromatinized elements, such as B chromosomes and other supernumerary chromosomal DNA fragments have long been known to be associated with asexuality in both plants and animals and are prime candidate regions for the evolution of multiple apomixis factors controlling the individual elements of apomixis. Here, we examined molecular evolution, gene regulation, and chromosomal location of a male apomeiosis factor (*UPG2*), a long noncoding RNA gene, in sexual and apomictic *Boechera* with and without male apomeiosis (i.e., balanced and unbalanced apomicts). We revealed the origin of the gene in the apomixis genome on an apomixis-specific, supernumerary heterochromatic *Boechera* chromosome (*Boe1*). The *UPG2* is active in the tapetum at male meiosis. We found allele classes specific to apomictic and sexual *Boechera* accessions and a third class that shares the features of both and points to a convergent transition state. Sex alleles are found only in some of the sexual accessions and have higher nucleotide divergence and lower transcriptional activity compared to apo alleles. These data demonstrate selective pressure to maintain the function of *UPG2* for unreduced pollen formation in apomicts as the occasional transmission of the allele from unbalanced apomicts into sexual organisms that lead to pseudogenization and functional decay of copies in sexual organisms.

## Introduction

The comparison of sexual and asexual organisms and their differences from the genetic (Hand and Koltunow, 2014) to the population levels (Smith, 1968; de Meeûs et al., 2007; Tomiuk, 2007) can be used as a model to elucidate the conditions (e.g., recombination rate, mutation frequency, linkage, and effect of ploidy) under which genes and their associated traits are formed *de novo* [cf. (Crow and Kimura, 1965)]. Relative to their sexual ancestors, it is important to differentiate between factors underlying the functional switch between both the modes of reproduction and those which have secondarily arisen under selection that favors asexual genotype fitness (Fyon and Lenormand, 2018). Apomixis, the asexual formation of plant embryos in seeds, has originated frequently within Angiosperms, and additionally demonstrates great phenotypic variability between different species [(Asker and Jerling, 1992), refer to review (Hojsgaard et al., 2014)]. Considering this, a one-size-fits-all rule for the genetic control of apomixis seems unlikely. Genetic analyses of some apomictic species (e.g., *Poa pratensis* and *Hypericum perforatum*) have pointed toward simple Mendelian inheritance of a few genes controlling the expression of apomixis which are tightly localized in euchromatic regions with normal genetic recombination [refer to reviews (Pupilli and Barcaccia, 2012) and (Barcaccia and Albertini, 2013)]. In other species (e.g., *Paspalum simplex* and *Pennisetum squamulatum*), genetic factors for apomixis reside in large non-recombining (i.e., heterochromatic) regions where they are typically accompanied by transposable elements, repetitive elements, and pseudogenes [refer to reviews (Pupilli and Barcaccia, 2012) and (Barcaccia and Albertini, 2013)]. From a functional perspective, apomixis could be viewed as a consequence of sexual failure [i.e., loss-of-function; cf. review (Barcaccia et al., 2020)], as has been shown by a loss of function leading to adaptation in multiple species (Monroe et al., 2021). Nonetheless, species-specific genetic regulation driven by independently-derived apomixis factors (Carman, 1997; Van Dijk and Vijverberg, 2005) and the absence of such factors in sexual relatives together point to apomixis as a gain-of-function trait (Vielle-Calzada et al., 1996).

Gain of apomixis function *via* the acquisition of new genetic elements could reflect (1) introgression through hybridization (Chapman and Bicknell, 2000; Kearney, 2005; Paun et al., 2006; Beck et al., 2011), (2) horizontal gene transfer (Yang et al., 2019), (3) a duplication-divergence scenario (e.g., pseudogenization), and/or (4) *de novo* gene origination from protogenes, such as intergenic regions, noncoding RNA with emerging open reading frames (ORFs) or overlapping gene ORFs (Andersson et al., 2015). In the two latter cases, novel gene functions require several hundred generations to evolve [cf. (Näsvall et al., 2012)]. Considering that varying portions of the genomes of apomictic plants (and parthenogenetic animals) are typically characterized by reduced recombination, supernumerary chromosome fragments (e.g., either in the form of B-chromosomes or intercalary segments on A-chromosomes), rearrangements, and non-homologous pairing during meiosis, this may provide an adaptive landscape within which new elements could arise [cf. *Pennisetum*, (Ozias-Akins et al., 1998); *Paspalum*, (Calderini et al., 2006); *Tripsacum*, (Grimanelli et al., 1998)].

In addition, apomictic genomes exhibit significant levels of hemizygosity [*sensu* Meselson effect; refer to (Brandt et al., 2021), also reviewed in (Hand and Koltunow, 2014)], and repetitive element accumulation (Calderini et al., 2006). Whether these structural genomic features are the cause or a consequence of apomixis is still a subject of debate (Hand and Koltunow, 2014). Understanding the origin and evolution of apomixis factors is further complicated by the fact that what appears to be linked factors in regions of reduced recombination can be segregated [cf. (Mau et al., 2021)].

The genus, *Boechera* Á. Löve & D. Löve (Boecher's rock cress; Brassicaceae), which is characterized by a basic chromosome number x = 7 and includes diploid sexuals (2n = 14) as well as diploid (2n = 14, 15) and triploid (2n = 21, 22) apomicts (Böcher, 1951; Schranz et al., 2005; Alexander et al., 2015), represents an ideal model system to resolve the evolution of apomixis by comparing sexual and apomictic reproduction at the genetic, cytogenetic, embryological, and molecular levels (Carman et al., 2019). Apomixis is gametophytic in *Boechera* with the *Taraxacum*-type of diplospory as a prevalent mode with some frequencies of the Hieracium-type apospory (Carman et al., 2019). Among apomicts, most lineages produce seeds with 2C:6C embryo-to-endosperm ratio (parthenogenetic egg cell development and fertilization of the central cell by unreduced pollen), but others produce seed with an “unbalanced” 2C:5C ratio by fertilization of the central cell with reduced pollen [*sensu* (Aliyu et al., 2010; Lovell et al., 2013; Mau et al., 2021)]. Rampant hybridization among diploid *Boechera* species has produced numerous diploid and triploid apomicts, which exhibit extensive morphological variation (Windham and Al-Shehbaz, 2006, 2007a,b) and many of these have become established as geographically and genetically distinct populations (Sharbel and Mitchell-Olds, 2001; Sharbel et al., 2005; Alexander et al., 2015). In apomictic *Boechera*, supernumerary heterochromatic DNA has been described in two forms, as B-like chromosome (*Del*) and as intercalary segments in an A-chromosome (*Het)* (Böcher, 1951; Sharbel et al., 2004, 2005; Kantama et al., 2007; Mandáková et al., 2015, 2020). In diploid *Boechera* apomicts (2n = 14), the *Het* chromosome was identified as a homolog of *Boe1* comprising blocks Aa, Ca, and D, with the expansion of pericentromeric heterochromatin (Mandáková et al., 2015). In aneuploid apomicts (2n = 15, 22), telocentric *Het'* (genomic blocks, Aa and Ca) and *Del* (block D) chromosomes originated through breakage within the heterochromatin-rich *Het* centromere (centric fission). The fission is reflected in the size of pericentric heterochromatin regions, which are larger on *Het'* than on *Het* and *Del* in 2n = 15 apomicts and it is hypothesized that accumulation of pericentric heterochromatin on the *Het* could have been an important prerequisite for the centric fission (Mandáková et al., 2015, 2020).

The identification of genetic factors associated with female [*APOLLO*; (Corral et al., 2013)] and male apomeiosis [*UPGRADE2* (*UPG2*); (Mau et al., 2013)], genome-wide changes in gene regulation in developing ovules (Sharbel et al., 2010), together with the segregation of all functional elements of apomixis (parthenogenesis, apomeiosis, and pseudogamy) and their variable intra- and interspecific transmission patterns (Mau et al., 2021) demonstrate that apomixis in *Boechera* is likely regulated by the inheritance of multiple factors controlling its various functional elements. Considering the frequent association between supernumerary chromatin and asexuality in plants and animals (Ozias-Akins et al., 1998), one possibility in *Boechera* is that the heterochromatic *Het, Het'*, and *Del* chromosomes serve as a sink for the synthesis, evolution, and preservation of genetic factors for apomixis.

In the current study, we traced the molecular evolution of the apomixis factor, *UPG2* in sexual and two types of apomictic *Boechera* (i.e., balanced and unbalanced) by assessing allele and promoter variation, its precise chromosomal localization, in addition to its spatial and temporal activity. We demonstrate that the *UPG2* gene is a prime example of how the reshaping of chromosomes by evolutionary forces, such as hybridization, has facilitated the formation of hotspots for the genetic control of apomixis.

## Materials and Methods

### Plant Material and Cultivation Conditions

Seeds from *Arabidopsis* and *Boechera* accessions (refer to Supplementary Tables 1, 11) were sterilized, cultured on an autoclaved MS media (4.3 g MS nutrient powder, bioPLUS™, GeneLinx International, Dublin/Ohio, USA; 8 g phytoagar, bioPLUS™, GeneLinx International, Dublin/Ohio, USA; pH 5.7 with 0.5 M NaOH) in sealed Petri dishes, and stratified at 4°C in the dark for 2 weeks. Seeds were transferred to short-day conditions (8h in light/16h in dark at 22°C) and germinated within 1–2 weeks. Seedlings were transferred into 1-inch pots with a substrate and grown 28 days (Sunshine® Mix #8/Fafard®-2; Sun Gro Horticulture, Vancouver, Canada). *Boechera* seedlings were vernalized in a cold chamber for 6 weeks at 4°C. All plants were transferred to four-inch square pots and grown under long-day conditions (17 h light and 21°C; 7 h dark and 18°C) at 350 μmol/m^2^/sec.

### Sequencing the *UPGRADE2* Gene and Its Promoter

The *UPGRADE2* gene (Mau et al., 2013) was amplified from 44 individuals of 12 sexual, 11 unbalanced, and 13 balanced *Boechera* accessions representing 13 *Boechera* species (Supplementary Table 1) by PCR with the primers, CON234X5-L (TCCGACCTAAATCCTACCAAACTGA) and CON234X5-R (TGCTCAATTTTGAACATCTTATTTGC) using the Phusion High-Fidelity DNA Polymerase (Cat#: F530L; Thermo Fisher Scientific, Waltham, MA, USA) following the manufacturer's specifications. All PCR products were gel-purified (NucleoSpin Plant II Mini kit; Macherey-Nagel, Düren, Germany) and cloned into *Escherichia coli* TOP10 cells using a TOP10 cells using the TOPO TA Cloning Kit for Sequencing (Cat#: 450071, Thermo Fisher Scientific, Waltham, MA, USA). Positive clones were selected by colony PCR using the T3 and T7 sequencing primers from the TOPO TA Cloning Kit for Sequencing (Supplementary Table 5). Individual clones were amplified using the TempliPhi™ DNA Sequencing Template Amplification Kit (Reagin, 2003) and Sanger sequenced on an ABI 3730 XL sequencing system using a set of external and internal primers (Supplementary Table 5).

The upstream promoter region of *UPGRADE2* [-1 and 3143nt in relation to the transcription start site (TSS)] from 8 sexual, 9 unbalanced, and 11 balanced *Boechera* accessions representing 12 *Boechera* species (Supplementary Table 1) was amplified by PCR using the primers, UPG2prom4F (CCCACGATTTTGGAACAATTC) and UPG2prom4R (GTTTGATTTCTCTACCTCCACAC) and the KOD Hot Start DNA Polymerase (Cat# 71086, EMD Millipore; Burlington, MA, USA) following the manufacturer's specifications. The PCR products were gel purified using the Wizard® SV Gel and PCR Clean-Up System (Promega, Madison, WI, USA), quantified with a Qubit 3.0 Fluorometer (Thermo Fisher Scientific, Waltham, MA, USA), and sequenced on a MiSeq system (Illumina; San Diego, CA, USA) with 100-fold coverage.

### Allele Analyses

Sequence reads were adapter- and quality-trimmed, assembled, and aligned into 55 *UPGRADE2* alleles from 36 *Boechera* accessions (refer to Supplementary Data Set 1) using the BBDuk Trimmer and CLUSTALW plugins of the Geneious software v2019.2.1 (Biomatters Limited; Auckland, New Zealand). Genetic diversity indices (Hd, π, and θ) for the *UPGRADE2* allele in reproductive groups (sexual, unbalanced, and balanced apomictic individuals) and Neutrality tests (Tajima's D and Fu's F^*^) were calculated with DnaSP V6 software (Rozas et al., 2017). The genetic heterogeneity statistics (Kxy, Gst, Δst, γst, Nst, Fst, Dxy, and Da) was calculated and genetic differentiation between the reproductive groups were tested by permutation (*n* = 10,000) and Chi-square tests. The gene flow (Nm) between reproductive groups was tested as a potential cause of changes in allele frequency between the reproductive groups. Analysis of the molecular variance (AMOVA) to calculate the source of the sequence variation (i.e., within or between reproductive groups) was estimated using Arlequin 3.5.2.2 software (Excoffier and Lischer, 2010). Polar haplotype sequence (i.e., alleles) trees of 55 *UPGRADE2* alleles from 36 *Boechera* accessions were constructed using the Mr. Bayes plugin of the Geneious software v2019.2.1 (Biomatters Limited; Auckland, New Zealand). The jModelTest v2.1.7 software (Posada, 2008; Darriba et al., 2012) selected the best nucleotide substitution model. Standard parameters were used with 1.1 million chain length of the Markov chain Monte Carlo and a burn-in length of 100,000.

Sequence networks of the *UPGRADE2* promoter region and the *UPGRADE2* gene were generated and visualized in R (R Development-Core-Team, 2019) using the package pegas (Paradis, 2010). Phylogenetic analysis was conducted separately for the *UPGRADE2* promoter and genic regions using MrBayes 3.2.7 (Ronquist et al., 2012) on CIPRES (Miller et al., 2012), applying the model, jumping approach for nucleotide substitution (lst = mixed) as well as gamma-shaped rate variation. After the removal of appropriate burn-in, two runs with two chains each were combined to create a maximum credibility consensus tree. Finally, hierarchical Bayesian analysis of population structure was carried out using the R package, rhierbaps (Cheng et al., 2013; Tonkin-Hill et al., 2018) and plotted onto the tree using the R packages, ape (Paradis et al., 2004) and phytools (Revell, 2012).

### Reverse Transcription Quantitative Real-Time PCR

For the quantitative polymerase chain reaction (qPCR), the SYBR® Green PCR Master Mix (Applied Biosystems, Foster City, CA) was used. The qPCR amplifications were carried out in a 7900HT Fast RT-PCR system machine (Applied Biosystems, Carlsbad, CA) with the following temperature profile for SYBR green assays: initial denaturation at 90°C for 10 min, followed by 40 cycles of 95°C for 15 s and 60°C for 1 min. For checking amplicon quality, a melting curve gradient was obtained from the product at the end of the amplification. The Ct, defined as the PCR cycle at which a statistically significant increase of reporter fluorescence is first detected, was used as a measure for the starting copy numbers of the target gene. The mean expression level and standard deviation for each set of three technical replicates for each complementary DNA (cDNA) was calculated. Relative quantification and normalization of the amplified targets were performed by the comparative ΔΔCt method using a calibrator sample in reference to the expression levels of the house-keeping gene, UBQ10 (Pellino et al., 2011).

Whole flowers at anther developmental stage 8 were collected from sexual and apomictic accessions for reverse transcription quantitative real-time PCR (RT-qPCR) analysis of the *UPGRADE2* gene. First strand cDNA was synthesized from four technical replicates per accessions according to the study by Mau et al. (2013). First, a non-allele-specific qRT-PCR analysis was performed on the same samples by using a pair of primers corresponding to a conserved region of exon 4, CON234B4-L (TTGCTTTGGTTGAATGCAATAC) and CON234B4-R (AATTACTAAATTTGCACACCACCTG; Supplementary Figure 2). Second, using the reverse primer, UPGALL-R (GGAAAGTCGACGGAAAAGAGCGTTT) in combination with two different forward PCR primers, UPG2SA-F (TCGTTCCTTGATTTTTTGTCGGAAACT) and UPG2AA-F (CGTTCCTTGATATTTAGTCGGATTTTTGT), which spanned one apoallele-specific polymorphism, it was possible to measure transcript abundance for both the apo- and sexalleles separately (Supplementary Figure 2A). The corresponding mean relative expression ratio for each genotype was calculated and significant differences between sex and apo alleles were evaluated using a Two-Factor ANOVA.

### *In silico* Analysis of Transcription Factor Binding Site Variation on the *UPG2* Promoter

For the identification of plant transcription factor (TF) binding motifs on the *UPG2* promoter, a consensus sequence of 3163nt upstream of the transcription start site (TSS; for *B. murrayi* x *stricta* ES524) from each 8 sexual, 8 unbalanced, and 11 balanced apomictic *Boechera* accessions was generated using default parameters in the Geneious software, v2019.2.1. Known TF binding sites (TFBSs) from *Boechera stricta* were mapped on the *UPG2* promoter consensus region using the Binding Site Prediction function of PlantRegMap [http://plantregmap.cbi.pku.edu.cn/; (Tian et al., 2019)] with the default *p*-value cut-off (≤1e-4). A Venn diagram (http://bioinformatics.psb.ugent.be/webtools/Venn/) of all mapped unique TFBSs on the selected *UPG2* promoter region was calculated to illustrate which TFBSs are specific or shared among the different reproductive modes. We tested for the overrepresentation of TF genes per TF family on the *UPG2pro* sequence in comparison with the *Boechera stricta* genome JGI(v1.2) (https://genome.jgi.doe.gov/) using the TF enrichment function of PlantRegMap [http://plantregmap.cbi.pku.edu.cn/; (Tian et al., 2019)]. The TFs were color coded by affiliation with a specific reproductive group and mapped onto the *UPG2pro* consensus sequences of the three reproductive groups using the Geneious software, v2019.2.1.

### Generation of *BoeUPG2* Promoter–β-Glucuronidase-Enhanced Green Fluorescent Protein Reporter Fusion and Arabidopsis Transformation

The upstream promoter region of *UPGRADE2* [-1 and 3143nt in relation to the transcription start site (TSS)] was amplified by PCR from the *Boechera* accession, ES514 using the primers, AttB1-UPG2-F (ggggacaagtttgtacaaaaagcaggcttCCCACGATTTTGGAACAATTC) and UPG2-R (CTTGCTCACCATCCGCGGGATATCCTGTGAAAGGGGATCGAGATTAGG; refer to Supplementary Table 5). The eGFP:GUS coding sequence including the 35s terminator was amplified by PCR from vector, pBGWFS7 (Karimi et al., 2002) using primers, eGFP-GUS-F (GATATCCCGCGGATGGTGAGCAAG) and t35s-attB2-R (ggggaccactttgtacaagaaagctggGTCACTGGATTTTGGTTTTAGG). The two amplicons were further combined by overlapping PCR using the primers, UP-AscI-F (ATTAGGCGCGCCCACGATTTTGGAACAATTC) and Pst-T35s-R (TTATCTGCAGTCACTGGATTTTGGTTTTAGG), then cloned to GateWay vector, pDONR221 for sequencing confirmation. The resulting cassette was subcloned to a modified version of pCAMBIA1300 vector (Ma et al., 2015) by AscI and PstI restriction enzymes. In order to facilitate transgenic screening with fluorescence, the Napin promoter=controlled dsRed cassette (Karimi et al., 2002) was amplified by Napin-PstI-F (ttatctgcagCATCGGTGATTGATTCCTT) and DsRed-Kpn-R (ttatggtaCCCGATCTAGTAACATAGATG), then inserted into the same T-DNA region.

These *pUPG2pro*::GUS-eGFP constructs were transferred into *Agrobacterium tumefaciens* strain, GV3101 to be used for Arabidopsis transformation in the absence of a functional protocol for *in vitro* transformation in *Boechera* at the time of the study. For transformation, Arabidopsis plants (ecotype Columbia) were grown in a growth chamber under long-day conditions (16 h/8 h light/dark cycle), temperature 23°C day/18°C night, light intensity 120–150 umol/m^2^ for 4–5 weeks and the bolts were cut. Approximately, 4–6 days after clipping, the plants were transformed by the floral dip method (Clough and Bent, 1998). Briefly, 20 ul of GV3101 Agrobacterium competent cell suspension and 1ul of *pUPG2pro*::GUS-eGFP plasmid were mixed and electroporated (1500 V, 25 uF, 200 M omega) in a 1 mm cuvette. The cell suspension was immediately transferred to 0.5 ml of SOC liquid medium and incubated at 28°C for 2 h, then spread onto YEP agar medium (for 1 liter add 10 g yeast extract; 10 g tryptone; 5 g NaCl; 1.2% agarose and adjust pH to 7.0) with 50 ug/ml kanamycin selection and incubated for 2 days at 28°C. Agrobacterium colonies were scraped from YEP agar plates and resuspended in the infiltration medium (1/2MS salts, 5% sucrose, 0.02% Silwet L77 Silwet L77; Lehle Seeds, Round Rock, TX) to an OD_600_ of 0.6 to 1.0. Plants were dipped in this suspension for 30 s with gentle agitation and placed 24 h in dark and humid conditions. Transformed plants were returned and grown in the growth chamber with the same condition for seed harvest. Fluorescent seeds, containing the transgene, were identified under a SteREO Discovery V12 Modular Stereo Microscope with a DsRED filter (Carl Zeiss Microscopy GmbH, Jena, Germany).

### Plant Genotyping

Genomic DNA was obtained from young leaves in all cases using a high-throughput method described by Mau et al. (2021). PCR was performed using the specific primers for eGFP (eGFP-F: ATGGTGAGCAAGGGCGAGGAG and Seq-eGFP-R: CGCCGGACACGCTGAACTTGTG; refer to Supplementary Table 1) and the Phusion U Green Hot Start DNA Polymerase (Thermo Fisher Scientific, Cat# F562L, Waltham, MA, USA) in a volume of 10 μL using 2 μl sample of DNA and 2.5 μM of each primer. The amplifications were run on a Mastercycler EP Gradient S (Eppendorf, Hamburg, Germany) under the following conditions: 3 min initial denaturation at 98°C; 40 cycles of amplification with 10 s at 98°C, 30 s at 54°C, and 1 min at 72°C; and 10 min of final elongation at 72°C. PCR success was verified with 1.5% agarose gel electrophoresis.

### β-Glucuronidase and Enhanced Green Fluorescent Protein Microscopy

Samples from Arabidopsis T2 plants for histochemical analysis of β-glucuronidase (GUS) activity were fixed in cold 90% (v/v) acetone on ice and subsequently incubated for 20 min at room temperature after all samples were collected. Then, tissues were washed twice in GUS staining buffer without X-Gluc (0.2 M of disodium phosphate, 0.2 M of monosodium phosphate, 0.1 M of potassium Ferrocyanide, 0.1 M of potassium Ferricyanide, 0.5M of EDTA disodium salt). The samples were stained for GUS activity in GUS staining buffer with 1.25 mM of 5-bromo-4-chloro-3-indolyl-β-D-glucuronide under vacuum for 20 min on ice and were incubated at 37°C overnight. Then the samples were washed twice in 70% (v/v) of ethanol and fixed overnight in 70% (v/v) of ethanol at room temperature. Chlorophyll- containing tissues were cleared in a solution of 1% of SDS and 0.2 N of NaOH overnight at room temperature and transferred into 70% (v/v) of ethanol, in which samples were stored at 4°C.

For visualization of the *UPGRADE2 promoter–GUS* activity, small flower buds from Arabidopsis T2 plants at anther developmental stages from the pollen of mother cell to the microspore formation (stages 5–9) were transferred to a clearing solution of 1% of SDS and 0.2 N of NaOH, and sepals and petals were partially removed to enhance the visibility of internal flower organs. The prepared flower bud was transferred into a drop of clearing solution on a microscope slide, covered with a cover slip, and directly used for visualization. Brightfield images were captured under 40× oil-immersion objective using an Olympus BX63 equipped with a DP80 dual CCD camera with Olympus cellSens Dimension software (Olympus, Tokyo, Japan). The Gus activity in developmental stages of larger flowers (i.e., tapetum degeneration and anthesis stage) was examined under brightfield conditions with a SteREO Discovery V12 Modular Stereo Microscope (Carl Zeiss Microscopy GmbH, Jena, Germany) and pictures were taken with the AxioVision 4.8 software (Carl Zeiss Microscopy GmbH, Jena, Germany). *UPGRADE2 promoter–GUS* activity in anthers was quantified in nine pUPG2pro::GUS-eGFP positive T2 plants from three independent transformation events in addition to each three 35s::GUS reporter lines and three lines negative for pUPG2pro::GUS-eGFP according to the study by Beziat et al. (2017).

Fresh whole flowers from three Arabidopsis T2 plants from each three independent transformation events were collected to visualize fluorescence due to BoeUPG2-promoter-driven enhanced green fluorescent protein (eGFP) expression. Smaller flower buds (developmental stages 5–9) were transferred to a clearing solution of 1% of SDS and 0.2 N of NaOH, and sepals and petals were partially removed to enhance the visibility of internal flower organs. Prepared flower buds were transferred into a drop of clearing solution on a microscope slide, covered with a cover slip, and directly used for visualization. Bright-field and fluorescence microscopy was performed using an Olympus BX63 equipped with a DP80 dual CCD camera under the epifluorescence channel for eGFP (U-MWB2 unit with BP460-490 nm excitation and BA520IF emission filter; Olympus, Tokyo, Japan). Images were captured under 40 × oil-immersion objective at a fixed exposure time of 500 ms in a dark room with Olympus cellSens Dimension software. Larger flower buds at tapetum degeneration stage and anthesis (stages 12 and 13) were dissected on a microscope slide, each two petals and sepals were removed, the flower buds were analyzed under bright-field and epifluorescence conditions with a SteREO Discovery V12 Modular Stereo Microscope (Carl Zeiss Microscopy GmbH, Jena, Germany), and pictures were taken with the software, AxioVision 4.8 (Carl Zeiss Microscopy GmbH, Jena, Germany).

### Chromosome Preparation and Fluorescence *in situ* Hybridization

Actively growing young root tips were collected from sexual (2n = 14; *B. crandalii* JL12, B15-1469), eudiploid apomictic (2n = 14; *B. murrayi x stricta* ES514, B15-2292), and aneuploid apomictic (2n = 15*; B. retrofracta x stricta* JL73, B12-693) *Boechera* plants cultivated in the greenhouse. Root tips were pre-treated with ice-cold water for 12 h, fixed in ethanol/acetic acid (3:1) fixative for 24 h at 4°C and stored at −20°C until further use. Chromosome spreads were prepared according to the published protocol (Mandáková and Lysak, 2016a).

Arabidopsis BAC contigs, corresponding to genomic blocks Aa (T7I23/At1g02300 - T25K16/At1g01010) and D (F1N19/At1g64700 - F14G9/At1g56140; The Arabidopsis Information Resource, TAIR2) were used as chromosome-specific painting probes following the chromosome structure of *Boechera* species (Mandáková et al., 2015). *Boechera* BAC clone F8G11 (Mau et al., 2013) was used for chromosome localization of *UPGRADE2*. Isolated BAC DNAs were labeled with Cy3-, biotin- or digoxigenin-dUTP by nick translation, as described by Mandáková and Lysak (2016b). The labeled probes were then pooled (150 ng of each Arabidopsis BAC and 600 ng of F8G11), mixed with unlabeled blocking DNA [6 μg of cleaved total genomic DNA of *B. stricta* ES06; (Mandáková et al., 2015)], ethanol-precipitated, desiccated, and dissolved in 20 μl of 50% of formamide and 10% of dextran sulfate in 2 × SSC per slide overnight. The probes were denatured together with chromosome-containing slides on a hot plate at 80°C for 2 min, hybridized overnight at 37°C, and washed in 20% of formamide in 2 × SSC at 42°C. The immunodetection of hapten-labeled probes was performed as described by Mandáková and Lysak (2016b).

After immunodetection, the preparations were stained with 4′,6-diamidino-2-phenylindole (DAPI; 2 μg/ml) in Vectashield (Vector Laboratories, Peterborough, UK). Fluorescence signals were analyzed using an Axioimager Z2 epifluorescence microscope (Zeiss, Oberkochen, Germany) and CoolCube CCD camera (MetaSystems, Newton, MA, USA). Images were acquired separately for the four fluorochromes using appropriate excitation and emission filters (AHF Analysentechnik, Tübingen, Germany). The three monochromatic images were pseudocolored and merged using Adobe Photoshop CS6 software (Adobe Systems, San Jose, CA, USA).

## Results

### *UPG2* Is Located on the Apo-Specific *Het* Chromosome

**Figure 1:**
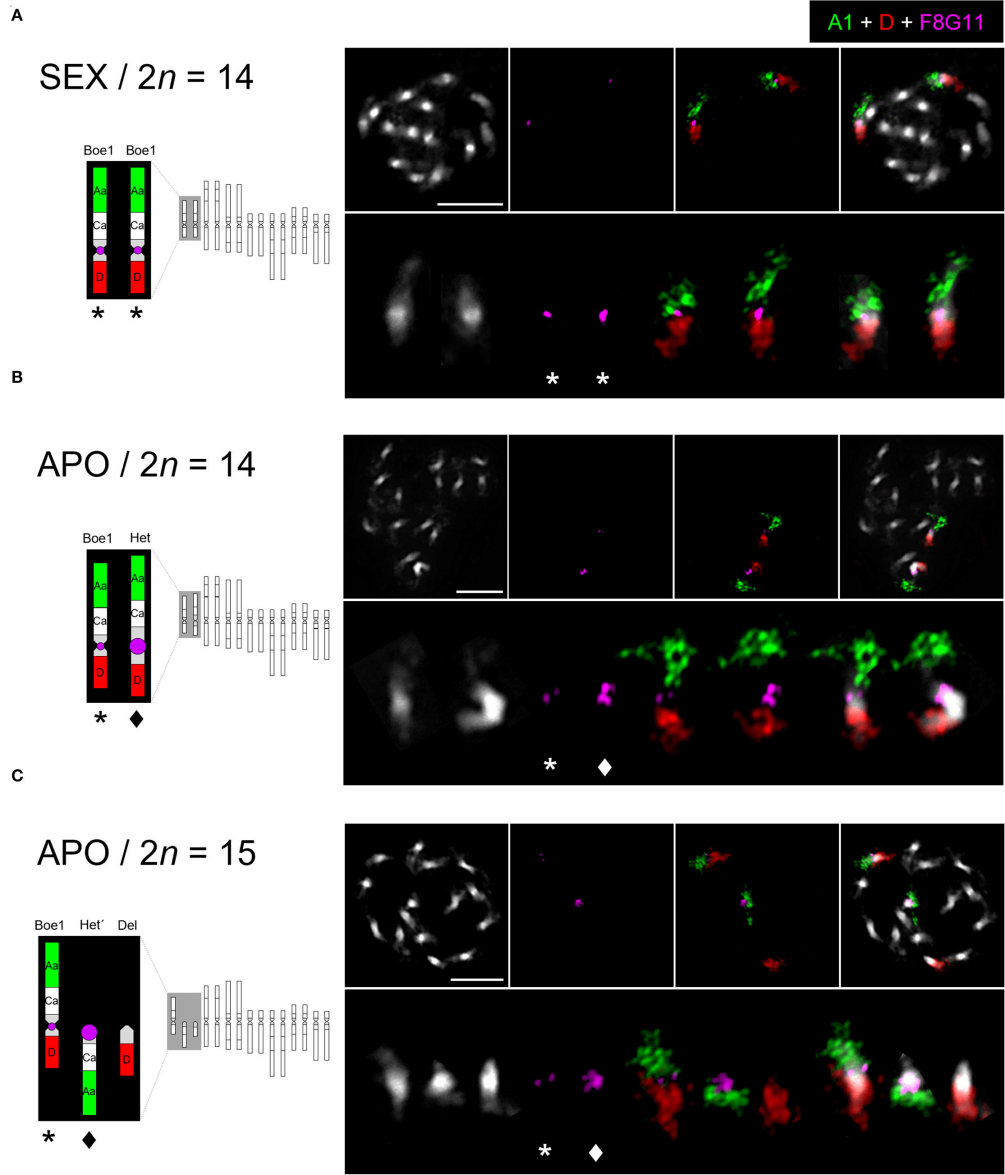
Chromosome localization of the *UPG2*-containing probe in sexual and apomictic *Boechera* accessions. FISH localization of the *UPG2*-containing *Boechera* BAC clone F8G11 and Arabidopsis BACs corresponding to genomic blocks Aa (green, T7I23/At1g02300 - T25K16/At1g01010) and D (red, F1N19/At1g64700 - F14G9/At1g56140) on mitotic chromosomes of a sexual (SEX / 2n = 14; *B. crandalii* JL12, B15-1469) **(A)**, eudiploid apomictic (APO / 2n = 14; *B. murrayi* x *stricta* ES514, B15-2292) **(B)**, and aneuploid apomictic (APO / 2n = 15; *B. retrofracta* x *stricta* JL73, B12-693) *Boechera* accessions **(C)**. Chromosomes were counterstained by DAPI; FISH signals are shown in color as indicated. Het (star) and Del (diamond), Scale bars, 10 μm.

Bacterial artificial chromosome (BAC)-based FISH was used to identify and reveal the structure of chromosomes *Boe1, Het, Het'*, and *Del* in diploid sexual (2n = 14; *B. crandalii* JL12, B15-1469), eudiploid apomictic (2n = 14; *B. murrayi* x *stricta* ES514, B15-2292) and aneuploid apomictic (2n = 15; *B. retrofracta* x *stricta* JL73, B12-693) *Boechera* accessions. Probes corresponding to genomic blocks, Aa and D were arranged according to Mandáková et al. (2015, 2020). In the sexual diploid, Aa and D marked the upper and bottom arms, respectively, of the two structurally identical homologs of *Boe1* (Figure 1A). In the eudiploid apomict, the *Het* chromosome was identified as a homolog of *Boe1*, with shared collinearity of genomic blocks as on *Boe1*, but with massive expansion of pericentromeric heterochromatin (Figure 1B). In the aneuploid apomict, the Aa and D probes hybridized on chromosome *Boe1* and two telocentric chromosomes, *Het'* (block Aa) and *Del* (block D; Figure 1C).

To demonstrate the direct link between *UPGRADE2 (UPG2)* and apomixis-related chromosomes (*Het, Het'* and *Del*), we performed FISH of the *UPG2*-containing BAC clone, F8G11 (Mau et al., 2013) on mitotic chromosome spreads. The *UPG2*-containing BAC hybridized to the pericentromeric region of chromosomes *Boe1, Het*, and *Het'*, with a significantly larger hybridization signal on *Het* and *Het'* in both apomictic forms but not to the centric end of the *Del* chromosome (Figures 1B,C). Thus, the data suggests a possible functional connection between the expansion of pericentromeric heterochromatin and amplification of *UPG2* gene copy number.

### Sexual and Apomictic *Boechera* Accessions Display Different *UPG2* Allele Classes

**Figure 2:**
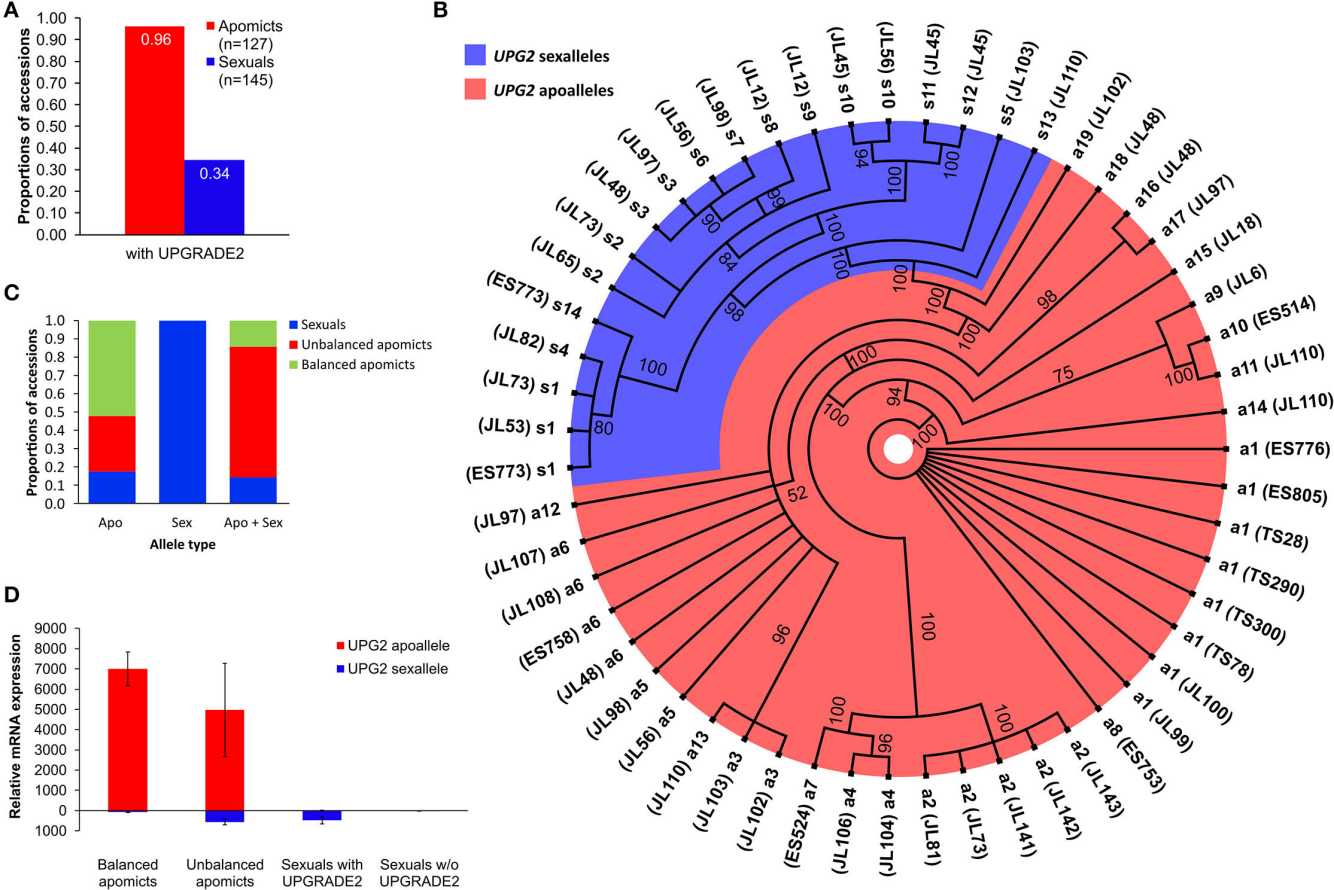
Sequence and expression variation of *UPG2* alleles in sexual, balanced, and unbalanced apomictic *Boechera* accessions. **(A)** Frequencies of *UPG2* in apomictic and sexual accessions flow cytometrically tested for seed ploidy. **(B)** The polar haplotype sequence tree of 55 *UPG2* alleles from 36 *Boechera* accessions was constructed using the Mr. Bayes plugin of the Geneious software v2019.2.1. The jModelTest v2.1.7 software selected the best model which was the Hasegawa-Kishino-Yano nucleotide substitution model with a proportion of invariable sites and a gamma-shaped distribution of rates across sites. Standard parameters were used with 1.1 million chain length of the Markov chain Monte Carlo and a burn-in length of 100,000. Scale bar indicates how much nucleotide change is reflected in the lengths of the branches. Different levels of polymorphism between alleles allow for the classification into “sex alleles” (blue shade) and “apo alleles” (red shade) due to their prevalent occurrence in sexual or apomictic accessions, which is supported by the analysis of the genetic population structure using BAPS5 (*cf*. cluster 3 in Figures 3A,B). Branch tips depict the different alleles and parentheses hold identifiers of sexual and apomictic accessions of *Boechera* (refer to Supplementary Table 2). **(C)** Various proportions of the allele types among sexual, balanced (i.e., pseudogamy with unreduced pollen), and unbalanced (i.e., pseudogamy with reduced pollen) apomictic accessions. **(D)** Quantitative reverse transcription-PCR analysis of allele-specific expression of the *UPG2* gene in flower bud tissue at meiosis. Distributions are based on the average of four technical replicates from each of four sexual individuals with *UPG2*, three sexual individuals without the gene, three unbalanced apomicts, and four balanced apomicts (refer to Supplemental Table 7). Different primer combinations were used for the detection of the apoallele (UPG2AA-F and UPG2ALL-R), sexallele (UPG2SA-F and UPG2ALL-R), and both alleles together (CON234B4_L and CON234B4_R; refer to Supplementary Table 5). The values are means calculated from Ct values of four technical replicates per sample (refer to Supplementary Table 6). Relative mRNA expression was normalized against tissue specific tested *Boechera* housekeeping genes, *ACTIN2* and *EF1*α.

We used *UPG2* genotyping data from 127 diploid apomictic and 145 diploid sexual *Boechera* plants for which the mode of reproduction was flow cytometrically analyzed [refer to Supplementary Dataset 1; (Mau et al., 2015)]. The *UPG2* gene is present in 34% and 96% of the tested sexual and apomict *Boechera*, respectively (Figure 2A).

**Figure 3:**
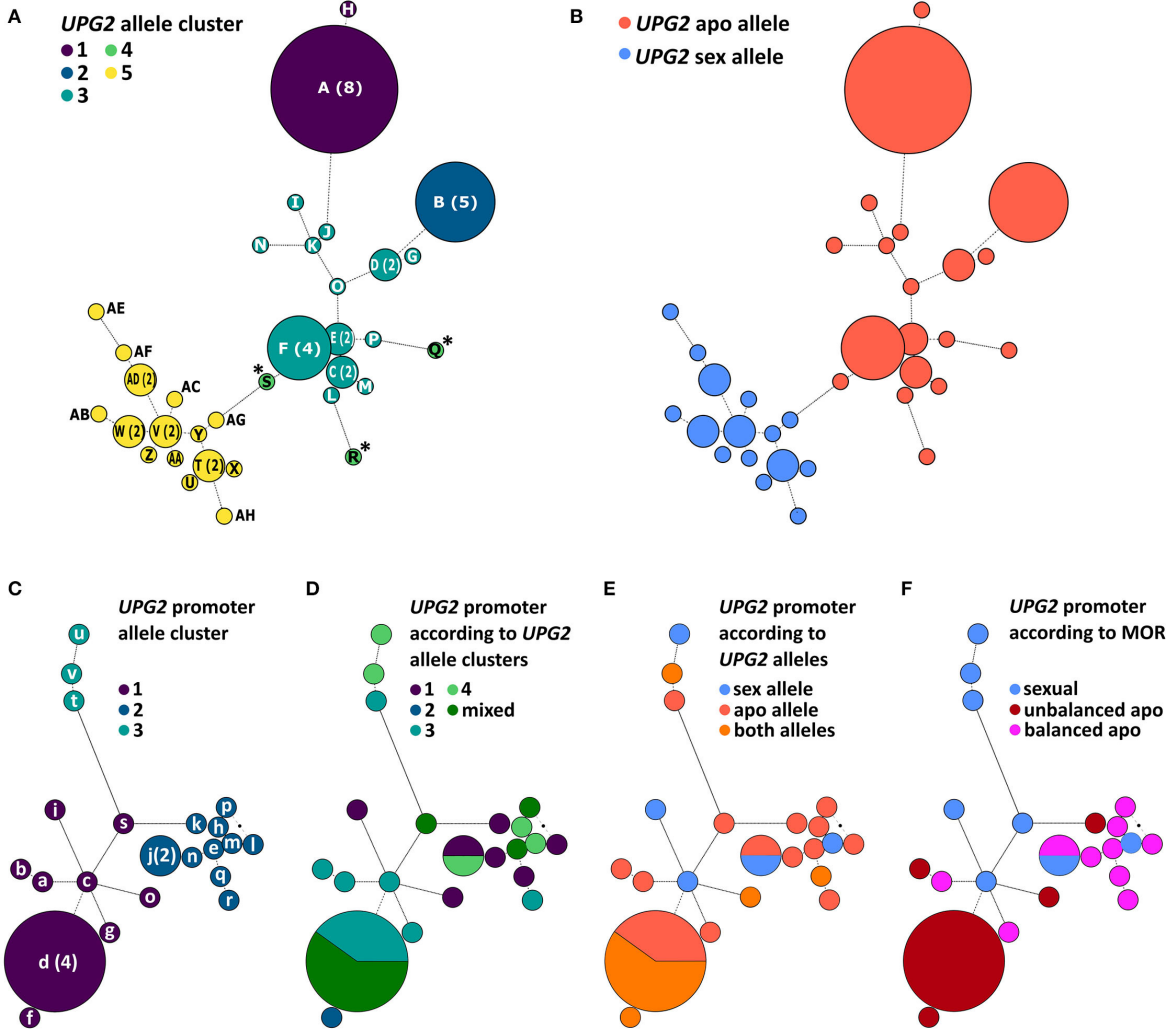
Phylogenetic analysis of the *UPG2* promoter and genic region in diploid sexual, balanced, and unbalanced apomictic *Boechera*. Allele networks of the *UPG2* gene **(A,B)** and the *UPG2* promoter region (from −1 – −3163 nt upstream of the transcription *UPG2* start site, **(C–F)** were generated by applying the model jumping approach for nucleotide substitution (lst = mixed) as well as gamma-shaped rate variation. A maximum credibility consensus tree was generated, and a hierarchical Bayesian analysis of population structure (BAPS) was carried out. Each leaf node of the network is labeled with a color corresponding to a BAPS cluster. The *UPG2* genic sequence was clustered into five groups **(A)** and majority assignment to reproductive modes of the accession enabled the classification of sex and apo alleles **(B)**. BAPS clustering of the *UPG2* promoter region led to three first-level allele clusters **(C)**. Overlays of *UPG2* BAPS clusters **(D)**, *UPG2* allele classes **(E)**, and mode of reproduction (MOR) of the tested *Boechera* accessions **(F)** onto the *UPG2* promoter sequence network were generated to test for the association of the covariates in the genic and promoter sequence network structure. Haplotype indices are generated in capital letters for the *UPG2* gene (A-AH) and in lower letters for the *UPG2* promoter sequences (a-v).

We tested the hypothesis that *UPG2* alleles from sexual *Boechera* represent a different class compared to alleles from unbalanced (i.e., parthenogenetic egg cell development and haploid pollen formation) or balanced apomictic *Boechera (*i.e., parthenogenetic egg cell development and unreduced pollen formation) by sequencing 3402 bp of the *UPG2* gene in 11 sexual, 12 unbalanced, and 13 balanced apomictic *Boechera* accessions (Supplementary Table 1). We identified 33 unique *UPG2* alleles which were classified into five clusters by hierarchical Bayesian analysis (Figure 3A, Supplementary Tables 1, 2). Alleles of clusters one to four are prevalently represented by apomicts (90.6%, min: 78.9%, max: 100%), whereas alleles of cluster five are predominant in sexuals (63.2%), thus distinguishing both “apo alleles” (*N* = 19) and “sex alleles” (*N* = 14; Figures 2B, 3B, Supplementary Tables 1, 2).

The sex alleles were strongly overrepresented in sexuals (63.6% of sexuals carried exclusively sex alleles or mixed sex and apo alleles, r_ϕ_ = −0.67, *p* = 0.0002; Fisher Exact test); while 58.3% of unbalanced and 92.3% of balanced apomicts carry only the apo alleles (r_ϕ_ = −0.45, *p* = 0.01; Fisher Exact test; Figure 2C). In addition, unbalanced apomicts carry both *UPG2* allele class more frequently (41.7%) compared to sexuals (9.1%) and balanced apomicts (7.7%, r_ϕ_ = −0.4, *p* = 0.029, Fisher Exact test; Figure 2C).

Interestingly, the sequences in cluster four (S, Q, and R, marked with asterisk; Figure 3A) were found only in unbalanced apomicts, and apparently emerged independently from cluster three, and their sequence identities were equidistantly low in each allele class (sequence identity within cluster four: average 95.2%, min 93.2%, max 98.0%; within apo allele class: average 98.5%, min 96.7%, max 100%; within sex allele class: average 98.9%, min 94.7%, max 100%; cluster four allele identity compared with apo allele class: average 95.8%, min 94.8%, max 96.7%; and with sex allele class: 95.9%, min 93.9%, max 97.5%; Supplementary Table 3). A sliding window analysis of the net nucleotide substitution per site (*D*_*a*_) across the *UPG2* gene sequence revealed that different regions of the alleles in cluster four diverge when independently compared to alleles of either the sex or the apo class (Supplementary Figure 1). For example, the center part of *UPG2* is conserved between sex and cluster four alleles while apo and cluster four alleles had a greater genetic distance (*D*_*a*_ at +1019-2407nt in sex vs. cluster four allele comparisons: average 0.0001, min −0.0037, max 0.0105; apo vs. cluster four allele comparisons: average 0.0049, min −0.0024, max 0.0147; Supplementary Figures 1D,G). In contrast, the genetic distance in both comparisons is reversed at the 5' end of *UPG2* (*D*_*a*_ at +2407-3204nt in sex vs. cluster four allele comparisons: average 0.0093, min −0.0006, max 0.0400; apo vs. cluster four allele comparisons: average −0.0006, min −0.0055, max 0.0014; Supplementary Figures 1D,G). Together, the data suggest that alleles S, Q, and R represent a group of alleles in transition between both allele classes.

### Genetic Differentiation Is the Greatest Among Apomictic Groups While Gene Flow Is the Highest Between Sexual and Unbalanced Apomictic Individuals

We investigated 33 unique alleles and 211 polymorphic sites of the *UPG2* sequence to test for genetic differentiation and gene flow between sexual, unbalanced, and balanced apomictic *Boechera* accessions (Supplementary Table 1). Diversity indices varied among the three reproductive modes (Supplementary Table 4). Sexual accessions showed the greatest haplotype diversity (*Hd* ± SD, 0.978 ± 0.027) and number of polymorphic sites (*S* = 162), while haplotype diversity was considerably lower in both, unbalanced (0.937 ± 0.033, *S* = 133) and balanced apomicts (0.800 ± 0.007, *S* = 124). Neutrality tests based on Tajima's *D* (Tajima, 1989) and Fu's *F*_S_ statistic (Fu, 1997), which give insights into demographic dynamics among members of the reproductive groups together were not significant, and point to the neutrality of the mutations (Supplementary Table 4).

Nucleotide divergence (*D*_*xy*_) and net genetic distance in nucleotides (*D*_*a*_) were comparably low between both apomictic groups (*D*_*a*_= 0.002, *D*_*xy*_= 0.015) and between sexual and unbalanced apomicts (*D*_*a*_= 0.003, *D*_*xy*_= 0.017), and the largest between sexual and balanced apomicts (*D*_*a*_= 0.006, *D*_*xy*_= 0.021; Supplementary Table 5). This is also reflected by the fixation indices (*F*_*ST*_ and *N*_*ST*_) which indicated the highest level of sequence differentiation between sexual and balanced apomictic individuals (*F*_*ST*_= 0.312, *N*_*ST*_= 0.314), whereas low levels of genetic differentiation exist between sexual and unbalanced apomicts and between both apomictic groups (*F*_*ST*_= 0.182, *N*_*ST*_= 0.183 and *F*_*ST*_= 0.125, *N*_*ST*_= 0.125, respectively; Supplementary Table 5). Genetic drift (*G*_*ST*_) and gene flow [*N*_*m*_, with Jukes and Cantor correction; Lynch and Crease (Lynch and Crease, 1990)] was detected between balanced and unbalanced apomictic *Boechera* (*G*_*ST*_= 0.049, *N*_*m*_= 1.75) and between sexual and unbalanced apomictic *Boechera* (*G*_*ST*_= 0.005, *N*_*m*_= 1.11; Supplementary Table 5). A stronger effect of genetic drift was detected between sexual and balanced apomictic *Boechera* (*G*_*ST*_= 0.049, *N*_*m*_= 0.55; Supplementary Table 5). The average within the group heterogeneity (*H*_*S*_) was significantly different between balanced and unbalanced apomictic accessions (*H*_*S*_= 0.884, χ^2^= 29.629, *p* = 0.041, *df* = 18) while no significant contrast was observed between the sexual and either of the apomictic groups (Supplementary Table 5).

The *UPG2* gene sequence was significantly genetically different between reproductive groups (*F*_*ST*_= 0.209, *p* < 0.001) with a low level of sequence variation (20.91%) compared to sequence variation within single groups (79.09%; locus by locus AMOVA; Supplementary Table 9).

### Allele Classes Differentially Expressed in Sexuals, Balanced, and Unbalanced Apomicts

We used allele-specific primers in a real-time quantitative reverse transcription (qRT)-PCR assay to test for the relative expression of both *UPG2* allele types in flower buds at meiosis of seven sexual, three unbalanced, and four apomictic individuals (Supplementary Figure 2A, Supplementary Tables 6, 7). The *UPG2* apo alleles were highly upregulated in flower buds from balanced and unbalanced apomicts, while no expression was detected for the apo allele in sexual individuals (Figure 2D, Supplementary Table 7). In contrast, while not expressed in balanced apomicts, the sex allele was transcribed at a lower level relative to the apo allele in unbalanced apomicts which harbor both allele types. The sex allele was comparably lowly expressed in sexual accessions and in unbalanced apomicts (Figure 2D, Supplementary Table 7). Overall, the sex allele was significantly lower expressed compared to the apo allele of the *UPG2* gene (*P* = 0.031, *df* = 1, *F* = 5.953, Two-Factor ANOVA).

### *UPG2* Promoter Sequence Variation Does Not Correlate With *UPG2* Alleles Classes

We tested the hypothesis that the divergent expression of sex and apo alleles of the *UPG2* gene is caused by a specific sequence variation in the *UPG2* promoter. We sequenced the 3151-bp region upstream of the transcription start site (TSS) of the *UPG2* gene in a subset of those used for the *UPG2* gene (i.e., 8 sexual, 10 balanced, and 9 unbalanced apomictic *Boechera* accessions) and compared the genetic population structure of the *UPG2* gene sequence with its *UPG2* promoter (Figures 3C–F, Supplementary Table 8). The *UPG2* promoter sequences were classified into three level-one Bayesian analysis of population structure (BAPS) clusters (Figure 3C). The overlay of sequence networks from *UPG2* promoter and *UPG2* gene showed that allele clusters from the gene were admixed and did not co-align with the sequence clusters of the *UPG2* promoter (Figure 3D). The comparison of the *UPG2* promoter allele clusters with the covariates, “*UPG2* allele class” (i.e., sex or apo allele) or “mode of reproduction” (MOR; i.e., sexual, unbalanced, or balanced apomictic) showed that none of the *UPG2* promoter allele clusters were exclusive to either covariate from the *UPG2* gene (Figures 3E,F, Supplementary Table 8). Overall, neither the allele classes nor the reproductive groups were reflected in the clustering of the *UPG2* promoter sequences.

The genetic diversity indices between the *UPG2* gene and its promoter are similar within each reproductive group (cf. Supplementary Tables 4, 5). Sexuals had the highest number of polymorphic sites (*N* = 243 in sexuals vs. 111 in unbalanced apomicts vs. 103 in balanced apomicts, respectively), haplotype diversity (1 ± 0.063 vs. 0.722 ± 0.159 vs. 0.978 ± 0.054), and nucleotide diversity (0.037 ± 0.005 vs. 0.010 ± 0.004 vs. 0.010 ± 0.004; Supplementary Table 4). Like the genic sequence, neutrality tests for the promoter region were not significant (cf. Supplementary Table 3). Nucleotide divergence (*Dxy*) and net genetic distance in nucleotides (*Da*) of the *UPG2* promoter sequence was comparable between all reproductive groups and higher compared to the genic sequence (cf. Supplementary Table 5). In contrast to the *UPG2* gene, the *UPG2* promoter showed the highest level of sequence differentiation between both apomictic groups (F_ST_ = 0.473, N_ST_ = 0.473), whereas a lower level of genetic differentiation was detected between sexuals and balanced (F_ST_ = 0.221, N_ST_ = 0.222) or unbalanced apomicts (F_ST_ = 0.284, N_ST_ = 0.285; Supplementary Table 5). The high level of *UPG2* promoter sequence differentiation between the apomictic groups was also reflected in a low level of gene exchange (G_ST_ = 0.075, Nm = 0.28), whereas considerable levels of gene flow between sexual and balanced or unbalanced apomictic *Boechera* were detected (G_ST_ = −0.005, Nm = 0.88 and G_ST_ = −0.006, Nm = 0.63, respectively; Supplementary Table 5).

The comparison of the *UPG2* gene and promoter sequences shows a decrease of sequence variation within reproductive groups (79.09–67.89%, respectively) while the variation between reproductive groups significantly increased (from 20.91 to 32.11%, respectively, F_ST_ = 0.321, *p* < 0.0001, locus by locus AMOVA; Supplementary Table 9).

### Sexual, Balanced, and Unbalanced Apomicts Share Most Binding Sites for Transcription Factors on *UPG2* Promoters

We tested for promoter motifs specific to *UPG2* promoter sequences in sexual, balanced, and unbalanced apomicts which could cause the divergent expression pattern of *UPG2* sex and apo alleles. In total, 516 transcription factor binding sites (TFBSs) from 262 TF genes were identified in the *UPG2* promoter region from −1 to −3163 nt from TSS in *B. murrayi x stricta* ES514. with similar numbers between the *UPG2* promoter in sexuals (420 sites), balanced (417 sites), and unbalanced apomicts (431 sites). The majority of TFBS are shared between the *UPG2* promoter sequences in sexuals, balanced, and unbalanced apomicts (340 of 516 TFBSs), and only a few are solely associated with *UPG2pro* sequences of a specific reproductive groups (Supplementary Figure 3, Supplementary Table 10). While balanced and unbalanced apomicts do not share specific TFBSs, the *UPG2* promoter sequences of sexuals and unbalanced apomicts share 21 TFBSs whereas sexuals and balanced apomicts share 51 TFBSs (Supplementary Figure 3, Supplementary Table 10). In comparison with all TF families identified in the *B. stricta* v1.2 genome (https://genome.jgi.doe.gov/portal/), we found that the Dof, ERF, Myb, and WRKY families were significantly overrepresented on *UPG2pro* (Supplementary Table 10). TFBSs were shared in *UPG2pro* sequences of sexuals, balanced, and unbalanced apomicts except for a few loci with more than one mapping. For example, a C2H2 binding site which is specific to unbalanced apomicts (position −2156 – −2177 bp from TSS), a MYB binding site specific to sexuals and balanced apomicts (−71– −101 bp from TSS), and a binding site for TFs from various families which is shared only among *UPG2* promoter sequences in sexuals and balanced apomicts (−1344– −1377 bp from TSS, Supplementary Figure 3).

Interestingly, almost half (24 of 53) of all mappings for the GAGA/CTCT-binding *BARLEY B RECOMBINANT / BASIC PENTACYSTEINE (BBR/BPC)* gene target the same *UPG2pro* region (from −1739 −1787 bp from TSS, Supplementary Figure 3).

### *UPG2* Promoter Activity Shifts From the Tapetum to the Style During the Flower Development in Arabidopsis

**Figure 4:**
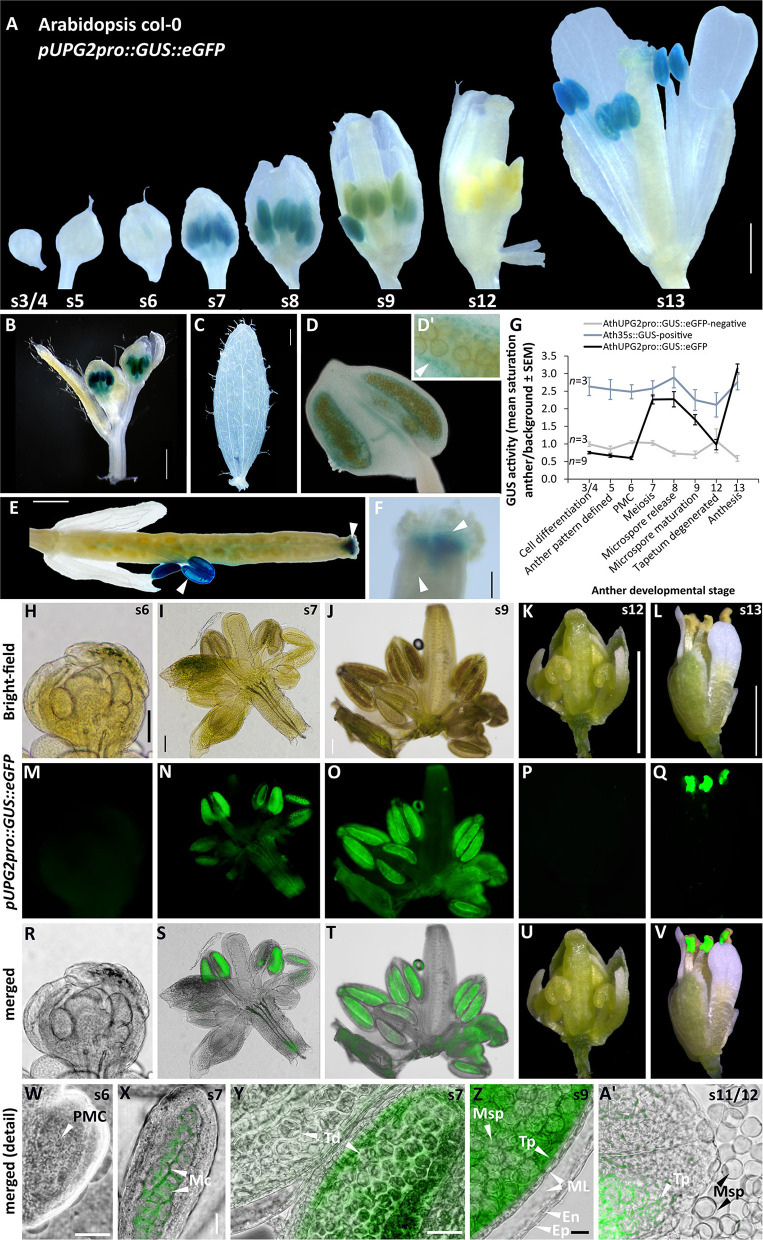
Temporal and spatial dynamics of the native apoallele, *Boechera UPG2* promoter–GUS:eGFP expression during Arabidopsis flower development and fertilization. Representative GUS fusion expression patterns of the native *UPG2* promoter from apomictic *Boechera* ES524 in Arabidopsis flowers with anther at cell differentiation [stage 3, cf. Sanders et al. (1999)], full differentiation (stages 4 and 5), pollen mother cell enlargement (stage 6), meiosis (stages 6 and 7), microspore release (stage 8), microspore maturation (stage 9), tapetum degeneration (stage 12), and anthesis (stage 13) **(A)**. In Arabidopsis, *UPG2* promoter–GUS expression was specific to anthers and absent in the stem **(B)** or leaf tissue **(C)**. The anther detail in **(D)** and its higher magnified region in **(D')** show *UPG2* promoter–GUS expression in the tapetum (indicated by arrow). In post-fertilization stage of flowers (anther stage 15), *UPG2 promoter–GUS* expression is indicated by arrows in the anthers **(E)** and in the style of the developing silique **(F)**. GUS activity in anthers was quantified [cf. Beziat et al. (2017)] in nine *pUPG2pro*::GUS-eGFP positive T2 plants from three independent in addition to each three 35s::GUS reporter lines and three lines negative for p*UPG2pro*::GUS-eGFP **(G)**. Bar = 0.5mm. The eGFP-*UPG2pro* was localized in Arabidopsis flowers with defined anther pattern and developed pollen in mother cells (stage 5, bar = 50μm), at meiosis (stage 6, bar = 50μm), at microspore maturation (stage 9, bar = 50μm), at tapetum degeneration (stage 12, bar = 1mm), and at anthesis (stage 13, bar = 1mm) using bright field images **(H–L)**, fluorescence of native *UPG2* promoter driven eGFP **(M–Q)** and merged images **(R–V)**. Merged bright field-eGFP epifluorescent microscopy images of anther locules of transgenic Arabidopsis plants expressing GFP-*UPG2* are shown **(W–A')**. GFP-*UPG2* was not detected in anthers at pollen mother cell stage but is visible in the tapetum at meiosis and during microgametogenesis. The GFP-*UPG2* signal fades at tapetum degeneration stage. Arrowheads point to flower organ structures: PMC, pollen mother cell; Mc, meiotic cell; Td, tapetum; Msp, microspore; ML, middle layer; En, endodermis; Ep, epidermis. Images show examples of three independent transgenic lines. Bars = 50 μm. 40 × oil-immersion objective, Olympus U-MWB2 unit with BP460-490nm excitation and BA520IF emission filter.

We have previously shown that the *UPG2* gene is specifically expressed in the anthers of apomictic *Boechera* at the onset of meiosis/apomeiosis (Mau et al., 2013). We performed an analysis of *UPG2* promoter (*UPG2pro*) activity in Arabidopsis to identify the specific spatial and temporal activity of the *UPG2* gene. We used a dual reporter GUS/eGFP gene construct placed under the regulation of a 3143-bp region upstream of the TSS of the *UPG2* gene from the apomictic *Boechera* accession ES514 (Supplementary Figure 2B). The GUS staining in different plant tissues at various developmental stages provided insights into the promoter activity dynamics (Figures 4A–F) and GUS activity was used to quantify promoter strength (Figure 4G). The *UPG2* upstream region actuated high levels of GUS activity in the anthers but did not cause GUS activity in other flower organs, leaves, or stem (Figures 4A–C). In the course of the anther development (Sanders et al., 1999), *UPG2pro* showed a bimodal activity pattern (Figures 4A,G). In early anther differentiation stages (anther stages 3–5) and during pollen mother cell development (anther stage 6), the *UPG2pro* was not active (Figures 4A,G). The *UPG2pro* activity started at meiosis (anther stage 7) in the anther locules (Figures 4D,D') and decreased during microspore maturation (anther stages 8 and 9), was not detected at the tapetum degeneration stage [(anther stage 12); Figures 4A,G], and triggered an unspecific GUS signal in the anther heads at anthesis stage (anther stage 13). Interestingly, during seed formation, the promoter showed activity in the style of green siliques in addition to its activity in anther heads (Figures 4E,F).

We further measured the *UPG2pro*-driven eGFP expression at the same flower developmental stages to visualize the tissue-specific localization of the *UPG2* gene (Figures 4H–A'). The GFP expression of *UPG2pro* was not detected during the development of mother cell pollen (anther stage 6) but emerged during meiocyte formation (anther stage 7) and was specific to the tapetum and not detected in any other anther tissue, such as epidermis, endodermis, or middle layers and was also not present in the microspores (Figures 4W–A'). The GFP expression was strong throughout meiosis (anther stage 7) and microsporogenesis (anther stage 8 and 9) and was absent at the tapetum degeneration stage (anther stage 12; Figures 4H–V). Remarkably, the GFP expression for *UPG2pro* recured in the anther heads at anthesis (anther stage 13; Figures 4L,Q,V).

## Discussion

### Heterochromatin Expansion in a Supernumerary Chromatin Potentially Provides Gene Space for the Origin of an Apomixis Factor

The chromosomal arrangement of sexual and asexual members of the genus, *Boechera* can greatly differ due to varying contributions of parental genomes in the hybrid apomicts, but the most prominent anomaly in apomicts is a largely heterochromatic chromosome (*Het*) which has been proposed to play a role in the genetic control of apomixis (Kantama et al., 2007). The *Het* and *Boe1* homologs have the same structure but differ by the expansion of pericentromeric heterochromatin on *Het* (Mandáková et al., 2015, 2020). Here, we show that the male-apomeiosis factor *UPG2* is localized in the pericentromeric heterochromatin of *Boe1* and its apo-specific homologs, *Het* and *Het'* (Figure 1). The *Het* homologs share many characteristics with heterochromatinized elements, such as B chromosomes and other supernumerary fragments, which have long been known to be associated with asexuality in both plants and animals (Camacho et al., 2000; Roche et al., 2001). In aposporous and diplosporous plant systems, supernumerary DNA was demonstrated to be a host for genomic regions associated with apomixis (Ozias-Akins et al., 1998; Kotani et al., 2014; Vašut et al., 2014). Here, for the first time, a functional apomixis gene has been demonstrated to be localized on such a highly heterochromatinized element in a plant species.

Heterochromatin domains differ in a number of features (e.g., gene density, chromatin landscape, and GC content) from euchromatic regions, but most peculiar is their association with the density of elevated transposable element (TE) and reduced recombination rates (Kent et al., 2017). Natural selection against the deleterious effects of TE insertions may lead to their transcriptional silencing and tolerance by the host genome in heterochromatin domains. In contrary to the broad-scale patterns of TE accumulation in low recombination domains, studies of fine-scale recombination rates have shown that regions of highly suppressed recombination in TE-rich clusters coexists adjacent to functional genes (Fu et al., 2002). The evolution of apomixis in *Boechera*, which includes the spread of apomixis factors *via* hybridization and associated disruptions to meiosis which accompany asexual seed formation, thus providing a mutation-selection context on the chromosomal level which facilitated the stepwise assembly of the highly chimeric and fragmented *UPG2* gene (Mau et al., 2013).

In support, we found a significantly stronger hybridization signal of the *UPG2*-harboring *Boechera* BAC clone F8G11 in the apo-specific *Het* and *Het'* compared with their homolog, *Boe1* (Figures 1B,C). These data suggest a possible functional connection between the expansion of pericentromeric heterochromatin (possibly caused by the accumulation of repetitive sequences) and amplification of the *UPG2* gene copy. Together with the absence of a specific promoter motif or sequence combination that consistently correlates with both *UPG2* alleles class, the higher transcriptional activity of the *UPG2* apo allele could be explained by increased gene copy number on the *Het* and *Het'* homologs. As expected, these evolutionary dynamics have led to deleterious mutation accumulation in apomictic *Boechera* [i.e., Muller's ratchet; (Lovell et al., 2017)], and one could imagine positive selection to maintain functional activity of genes found in such heterochromatic genome regions. There, variation in copy number can influence a gene's expression level through the number of functional copies or by position effects, for example in *cis*- and *trans*-gene regulatory sequences (Zmieńko et al., 2014).

Hence, the *Het* homolog could act as a sink for an apomixis locus comprising additional factors across a larger chromosomal span. In other apomictic plants, apomixis loci are often located in degenerate hemizygous chromosome regions which do not recombine with the ancestral sexual chromosome homolog (Ozias-Akins et al., 1998; Underwood et al., 2022). In the same light, asymmetric heterochromatin expansion on the divergent *Het* in *Boechera* could provide a mechanism (e.g., TE insertions) whereby beneficial mutations (i.e., here factors controlling apomixis) are driven to higher frequency [i.e., Hill–Robertson interference; (Kent et al., 2017)].

### *UPG2* Is a Potential Tapetal Regulator of Microspore Development at Meiosis

The tapetum is a one-cell layer tissue which is in direct contact with the sporogenous tissue and provides necessary enzymes and nutrients which play a crucial role in the development and maturation of microspores (Scott et al., 2004). Growing evidence emerges that a cell-to-cell communication between both cell types exist and that small RNAs, specifically microRNAs and associated regulators that are localized in the tapetum play an important role in the regulation of male meiosis, although the exact role of the tapetum is not yet clear [cf. review by (Lei and Liu, 2020)]. In this context, the *UPG2* gene could be an interesting candidate as it represents a long non-coding primary microRNA (pri-miRNA) gene that potentially emits multiple miRNAs (Mau et al., 2013). Its specific activity in the tapetal cell layer at the onset of meiosis with a subsequent loss of activity during the programmed tapetal cell death together point to a prominent role in guarding the development of pollen during meiosis.

Both reporter genes (GUS and GFP) confirmed a bimodal expression pattern throughout anther development, with (1) tapetum-specific expression pattern during male meiosis, (2) unspecific expression in all anther tissues during anthesis, and (3) style-specific activity at silique maturation (Figure 4). One potential explanation for the spatiotemporal shift in the activation of *UPG2pro* during flower maturation is that *UPG2pro* is controlled by a set of upstream regulatory elements, such as enhancers, silencers, and transcription factors (TFs), which is unique to the different floral tissues at a given stage throughout flower maturation. Consistent with our findings, four overrepresented TF families (Myb, Dof, ERF, and WRKY) among a total of 34 TF families binding to 516 TF binding sites (TFBSs) on the *UPG2* upstream sequence have important functions in the transcriptional regulation of a variety of biological processes related to growth and development, and have been shown to have different temporal and spatial expression patterns (Cao et al., 2020). It is unclear whether tapetum-specificity of *UPG2pro* is facilitated by the *BARLEY B RECOMBINANT/BASIC PENTACYSTEINE (BBR/BPC)* TF family, which was most abundant in terms of mapping to *UPG2pro* (Supplementary Figure 3A), but support is provided by the specific role of *BBR/BPC* members in the control of flower and seed development (Theune et al., 2019).

### Signs of Positive Selection on *UPG2* Apo Alleles in Apomicts, but Not in Sexuals

The identification of apomixis factors which are often unique to their plant model [reviewed in (Schmidt, 2020)] leads one to question their origin, evolution, and patterns of maintenance in natural apomictic populations. We thus compared the levels of allelic variation of an apomixis gene between reproductive modes to find evidence for (i) gene decay and/or specific variants that can influence function, (ii) gene flow between reproductive groups, and (iii) specific allele variants showing signs of selection.

*UPG2* alleles in sexual accessions show a higher level of haplotype diversity compared to either unbalanced and balanced apomictic accessions (Supplementary Tables 4, 5). Prevalent hybridization between sexuals and unbalanced apomicts occur in *Boechera* (Mau et al., 2021), with multiple segregating apomixis factors leading to the establishment of *de novo* apomictic lineages in addition to “failed apomictic lineages” in which the remnants of sexual reproduction can still be found (Mau et al., 2021). The observed allele variation of *UPG2* across the three reproductive modes supports the hypothesis that while the majority of sexuals are deficient of *UPG2* alleles, occasional copies of *UPG2* are transferred to sexuals *via* hybridization with unbalanced apomictic donors (Mau et al., 2021), but fail to create *de novo* apomictic lineages since not all needed factors were inherited. Subsequently, the lower transcriptional activity and higher haplotype diversity and the number of polymorphisms in sexual *UPG2* alleles point to some degree of functional gene decay (i.e., pseudogenization) that eventually is caused by an absence of selection to maintain function during meiosis in sexual plants.

Unbalanced apomicts often carry sex and apo allele classes whereby the sex alleles might have been reintroduced by sexuals which carry the *UPG2* allele. This is supported by a greater gene flow, a lower genetic divergence, and a higher level of seed set in hybrids between sexuals and unbalanced apomicts compared to those between sexuals and balanced apomicts were observed (Mau et al., 2021). Together, this reflects a trend toward isolation between balanced apomicts and sexuals [*sensu* Muller's ratchet, refer to (Lovell et al., 2017); Supplementary Tables 4, 5]. Interestingly, the relative contributions of the allele classes in conjunction with yet unknown gene regulatory factors could define a titer of the overall transcriptional activity of the *UPG2* gene (cf. Figure 2D) and thus explain the facultative expression of the reduced pollen phenotype in all tested unbalanced apomicts [i.e., they still produce varying frequencies of unreduced pollen; (Aliyu et al., 2010; Mau et al., 2021)].

The genetic diversity at the *UPG2* locus is comparable between sexual and both apomictic reproductive groups, in addition to between apomictic reproductive groups (Supplementary Table 4). Ongoing hybridization between forms in natural populations *via* haploid pollen [i.e., cluster 4 alleles; Figure 3A and Supplementary Figures 1D,G; (Mau et al., 2021)] could explain similar levels of diversity, which supports theories explaining the paradox whereby the spread of apomixis to fixation in a population is not necessarily associated with a strong decrease of genetic variability (Adolfsson and Bengtsson, 2007). Thus, unbalanced apomictic populations, which mostly contain both *UPG2* allele classes, could have served as incubators for the evolution of novel allele variants by accumulating mutations that may slightly modify and fine-tune sub-traits of apomixis, such as male apomeiosis. Finally, due to their haploid pollen function they also continue to serve as key distributors making the favorable allelic variants available in a multitude of genetic backgrounds [*sensu* (Adolfsson and Bengtsson, 2007)].

**Figure 5:**
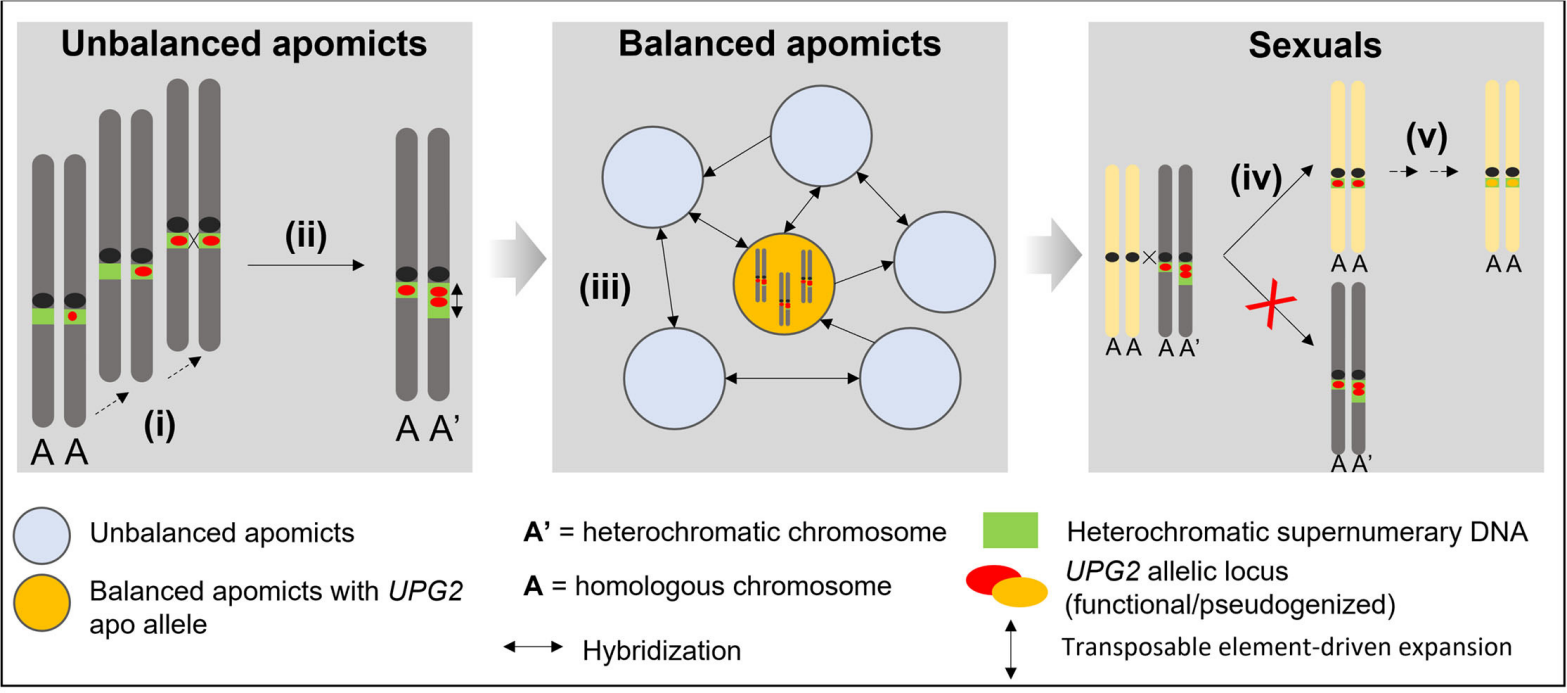
Conceptual model for explaining the stepwise evolution of an asexual allele class for haploid pollen formation in supernumerary DNA in *Boechera*. (i) Convergent transposable element-driven stepwise *de novo* synthesis of apomictic alleles in unbalanced apomicts; (ii) Functional preservation through asymmetric heterochromatin expansion and asynapsis; (iii) Hybridization-driven reticulation *via* haploid pollen from unbalanced apomicts and positive selection of apo alleles to maintain full functional activity in balanced apomictic populations; (iv) Hybridization-driven transmission into some sexual individuals and failure to produce apomictic progeny; (v) Functional degradation and pseudogenization.

The observations here on *UPG2* shed light upon evolutionary steps gain-of-function factors for asexuality in *Boechera* may have undertaken in general (cf. Figure 5). It is likely that the fragmented composition of *UPG2* alleles must have been caused by a stepwise evolutionary process, and its location in supernumerary heterochromatic DNA together with our discovery of alleles in transition between apo alleles and sex alleles in some unbalanced apomicts point to unbalanced apomicts as the origin for the *de novo* synthesis of apomictic *UPG2* alleles (Figures 3A, 5). Eventually those apomicts carrying the novel allele class could preserve its beneficial function through an absence of synapsis and duplication in the expanding heterochromatin. The high levels of gene flow between sexuals and unbalanced apomicts in conjunction with the previously detected infectious transmission of apomixis factors through haploid pollen (Mau et al., 2021) have probably led to the appearance of *UPG2* alleles in some sexuals but failure to initiate full apomixis in these plants have led to functional degradation and pseudogenization of *UPG2* in the sexual lineages. Recurrent hybridization of unbalanced apomicts play an important role in the spread of discrete apomixis factors frequently forming balanced apomictic progeny (Figure 5).

On a practical note, genetic analyses of apomixis have revealed that many candidate genes are associated with spatial and temporal variability of expression profiles in reproductive tissues contributing to a temporal shift (heterochrony) in gametophyte and embryo development between sexual and apomictic reproduction (Carman, 1997; Sharbel et al., 2010). Therefore, the identification, isolation, and validation of appropriate time and organ/cell-specific promoters, such as *UPG2*, is a prerequisite for the modification of a defined set of functions to induce apomixis for breeding purposes without causing side or off target effects.

## Data Availability Statement

The datasets presented in this study can be found in online repositories. The names of the repository/repositories and accession number(s) can be found in the article/Supplementary Material.

## Author Contributions

MM and TS conceived of the concepts. MM, XM, TM, and ML designed the experiments. MM, XM, LZ, and TM performed the experiments. MM, JE, and TM analyzed the data. MM, TM, ML, and TS wrote the manuscript. All authors contributed to the article and approved the submitted version.

## Funding

This work was co-funded by a grant from the Global Institute of Food Security at the University of Saskatchewan to TS and the operational funds of TS at the Leibniz Institute for Plant Genetics and Crop Plant Science (IPK Gatersleben). This work was supported by the Czech Science Foundation (project no. 21-06839S).

## Conflict of Interest

The authors declare that the research was conducted in the absence of any commercial or financial relationships that could be construed as a potential conflict of interest.

## Publisher's Note

All claims expressed in this article are solely those of the authors and do not necessarily represent those of their affiliated organizations, or those of the publisher, the editors and the reviewers. Any product that may be evaluated in this article, or claim that may be made by its manufacturer, is not guaranteed or endorsed by the publisher.
