# Supplementary material for: Evolution of an Apomixis-Specific Allele Class in Supernumerary Chromatin of Apomictic Boechera
Source: Front Plant Sci. 2022 Jun 1;13:890038. doi: 10.3389/fpls.2022.890038 (PMC9198585; doi:10.3389/fpls.2022.890038)
Supplement: Supplementary file 1 [file Data_Sheet_1.docx]

Supplementary Material

# Supplementary Figures


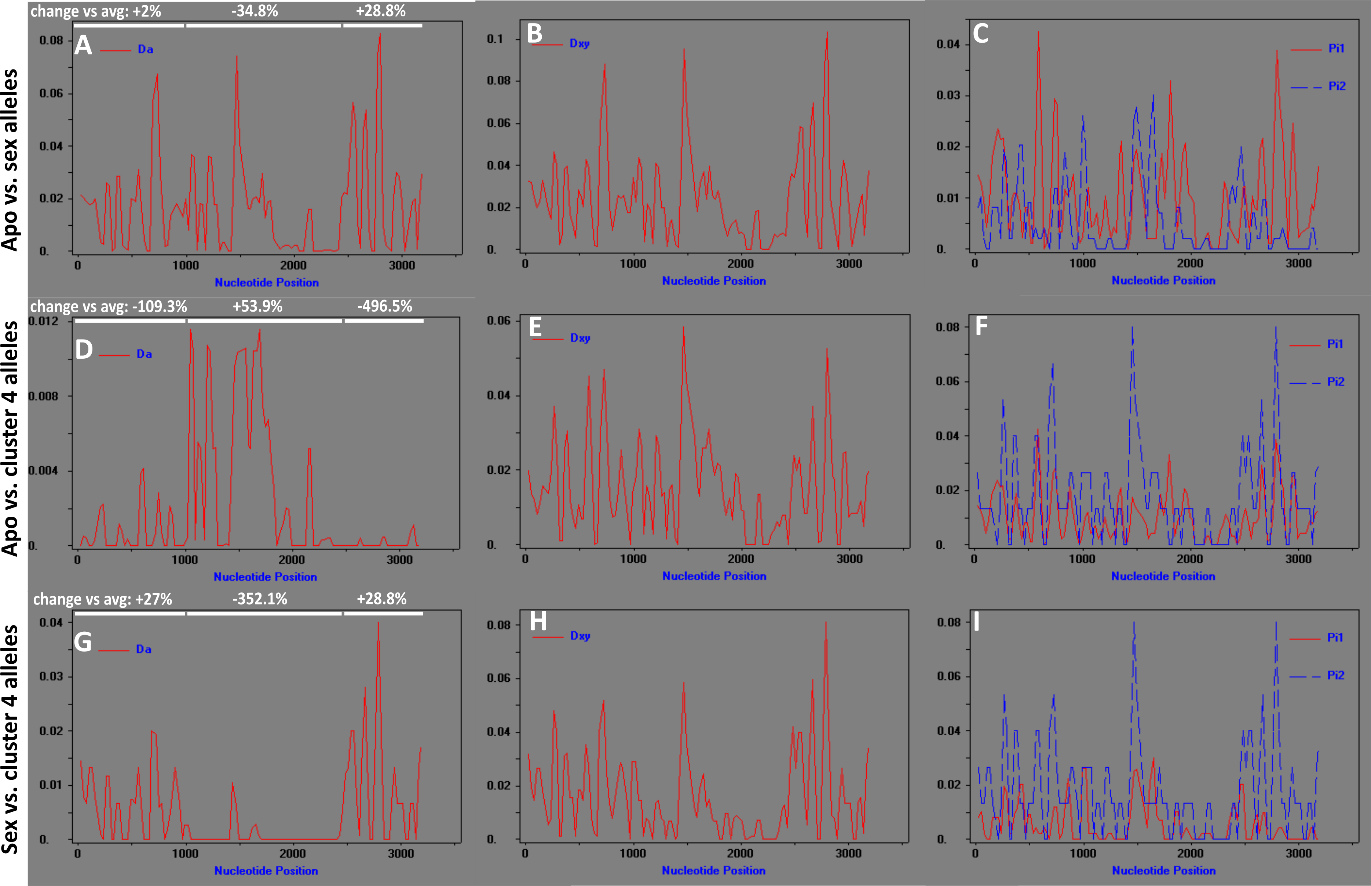


**Supplementary Figure 1.** Sliding window analysis of sequence divergence between sex alleles, apo alleles and BAPS cluster 4 alleles of the *UPG2* gene in sexual, balanced and unbalanced apomictic *Boechera* accessions. We analysed 55 *UPG2* alleles of 2936bp length from 36 *Boechera* accessions with a sliding window approach (window lengths: 50 bps, step size: 25 bps) to compare the nucleotide substitution per site (Dxy), the net nucleotide substitution per site (Da) and the nucleotide diversity (π) between sex and apo alleles (A-C), between apo alleles and alleles of the BAPS cluster 4 (D-F), and between sex alleles and alleles of the BAPS cluster 4 (G-I).


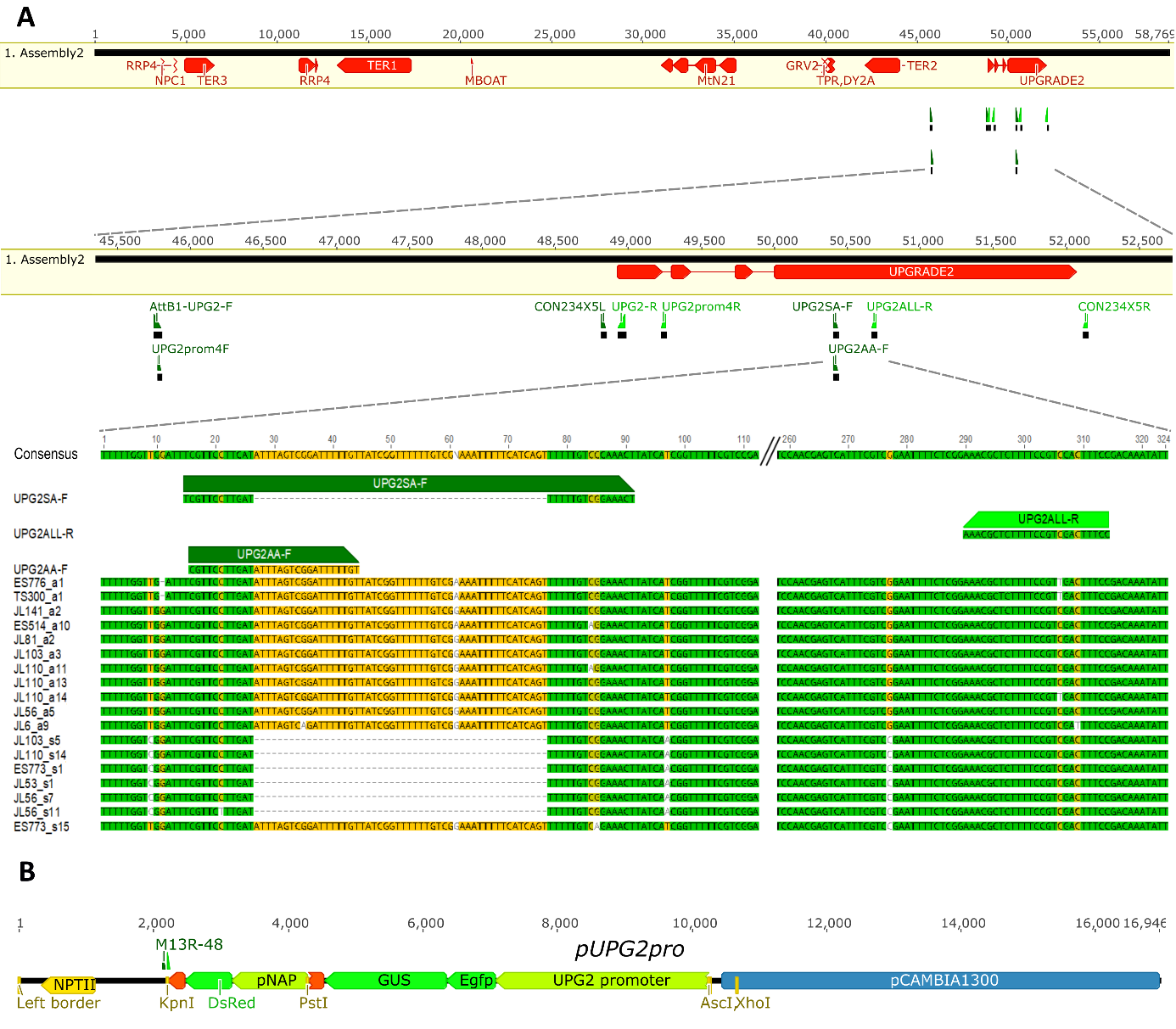


**Supplementary Figure 2.** Primer setup for phylogenetic and RT-qPCR analyses of *UPGRADE2* alleles and schematic representation of the *UPG2* promoter-reporter construct. Primers were designed using the *UPG2* locus BAC clone assembly from Mau et al. (2013) (A). Sex and apo-specific *UPG2* alleles were determined by phylogenetic analyses of the total *UPG2* genomic sequence. The transcript abundance for both alleles with RT-qPCR was measured using the reverse primer UPG2ALL-R and two different forward primers, UPG2SA-F and UPG2AA-F which spanned the most common apo-specific polymorphism (ATTTAGTCGGATTTTTGTTATCGGTTTTTTGTCGGAAATTTTTCATCAGT, position in *UPG2* genomic sequence: 1507nt - 1557nt). The allele s15 in *Boechera* accession ES773 is a single outlier to the allele assignment into sex- and apoallele. The expression vector pUPG2pro was generated by subcloning the *UPG2* promoter region between -3’141bp and +35nt relative to the *UPG2* transcription start site and the eGFP:GUS coding sequence including the 35s terminator into a modified pCAMBIA1300 vector with the RedSeed selection marker (pNAP::DsRed) (B).


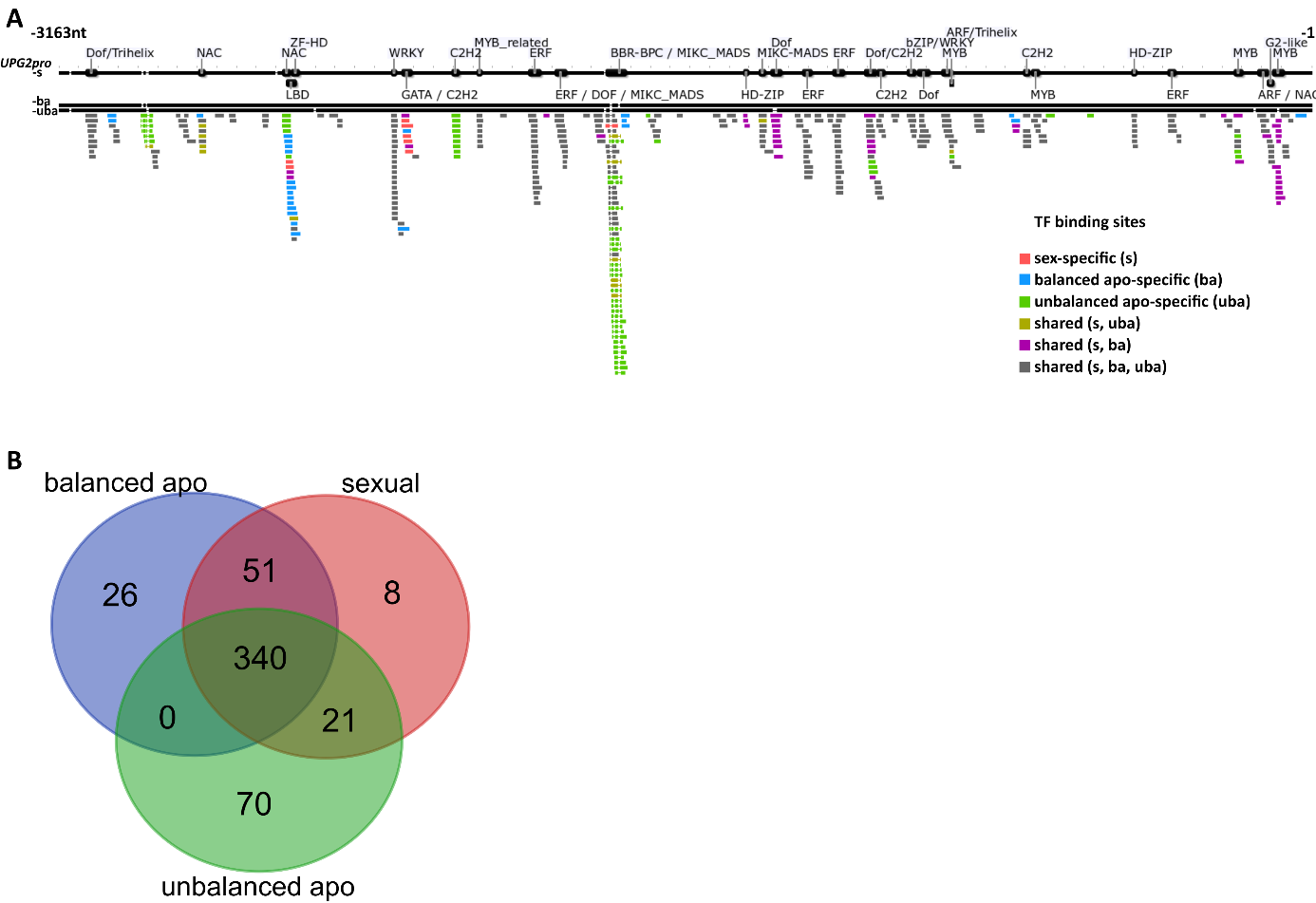


**Supplementary Figure 3.** Transcription factor (TF) binding prediction on *UPG2* promoter region in sexual, balanced apomictic and unbalanced apomictic *Boechera* accessions. Potential binding sites for transcription factors from a sexual *Boechera stricta* genome (http://phytozome.jgi.doe.gov/) on the *UPG2* promoter consensus region (-1 to -3143 in *B. murrayi x stricta* ES514) from 8 sexual, 11 balanced and 8 unbalanced apomictic *Boechera* accessions were identified with the Binding Site Prediction function of the PlantRegMap software (<http://plantregmap.cbi.pku.edu.cn/>; [1]; p-value cut-off ≤1e-4). In total, 516 unique TFBSs were identified across the *UPG2* promoter consensus sequences from sexuals, balanced and unbalanced apomicts, and loci with more than five mapped reads are depicted on the reference consensus sequence (A). The Venn diagram (<http://bioinformatics.psb.ugent.be/webtools/Venn/>) shows that *UPG2* promoters in sexuals, balanced and unbalanced apomicts share 340 from 516 unique TFBSs (A).

# Supplementary Tables

| **Table S1.** Occurrence of sex and apo allele types of the *UPGRADE2* gene in diploid sexual and diploid balanced and unbalanced apomictic *Boechera* accessions. | | | | | | | | | | |
| --- | --- | --- | --- | --- | --- | --- | --- | --- | --- | --- |
| **#** | **Accession*** | **Taxon*** | **Mode of reproduction^†^** | **Seed ploidy^‡^** | **No. of individuals tested** | **No. of clones sequenced** | | ***UPG2* sex allele^§^** | ***UPG2* apo allele^§^** | **Experiments*** |
| **1** | JL56 | *Boechera fernaldiana* | Sexual | 2C:3C | 1 | 8 | **s6, s10** | | **a5** | **1, 3** |
| **2** | JL81 | *B. stricta* x *retrofracta* | Sexual | 2C:3C | 1 | 10 | **-** | | **a2** | **1** |
| **3** | JL65 | *B. stricta* | Sexual | 2C:3C | 1 | 12 | **s2** | | **-** | **1, 2** |
| **4** | JL6 | *B. pallidifolia* | Sexual | 2C:3C | 2 | 27 | **-** | | **a9** | **1, 2, 3** |
| **5** | JL18 | *B. pallidifolia* | Sexual | 2C:3C | 1 | 9 | **-** | | **a15** | **1, 2** |
| **6** | JL12 | *B. crandallii*^††^ | Sexual | 2C:3C | 1 | 5 | **s8, s9** | | **-** | **1, 2, 4** |
| **7** | JL53 | *B. crandallii*^††^ | Sexual | 2C:3C | 3 | 31 | **s1** | | **-** | **1, 2, 3, 4** |
| **8** | ES773 | *B. crandallii* | Sexual | 2C:3C | 2 | 24 | **s1, s14** | | **-** | **1, 2, 3** |
| **9** | ES758 | *B. glareosa* | Sexual | 2C:3C | 1 | 12 | **-** | | **a6** | **1, 2** |
| **10** | JL45 | *B. oxylobula* | Sexual | 2C:3C | 1 | 7 | **s10, s11, s12** | | **-** | **1, 2** |
| **11** | JL82 | *B. stricta* x *retrofracta* | Sexual | 2C:3C | 1 | 7 | **s4** | | **-** | **1** |
| **12** | JL48 | *B. crandallii* x *thompsonii*^††^ | Unbalanced apomict | 2C:5C | 1 | 7 | **s3** | | **a6, a16, a18** | **1, 2, 4** |
| **13** | JL97 | *B. pallidifolia* | Unbalanced apomict | 2C:5C | 1 | 7 | **s3** | | **a12, a17** | **1, 2** |
| **14** | JL98 | *B. pallidifolia* | Unbalanced apomict | 2C:5C | 1 | 8 | **s7** | | **a5** | **1, 2** |
| **15** | JL107 | *B. stricta* | Unbalanced apomict | 2C:5C | 1 | 15 | **-** | | **a6** | **1, 2** |
| **16** | JL108 | *B. stricta* | Unbalanced apomict | 2C:5C | 1 | 10 | **-** | | **a6** | **1, 2** |
| **17** | JL102 | *B. crandallii* | Unbalanced apomict | 2C:5C | 1 | 4 | **-** | | **a3, a19** | **1** |
| **18** | JL103 | *B. crandallii* | Unbalanced apomict | 2C:5C | 2 | 20 | **s5** | | **a3** | **1, 2, 3** |
| **19** | JL99 | *B. retrofracta* | Unbalanced apomict | 2C:5C | 1 | 17 | **-** | | **a1** | **1, 2** |
| **20** | JL100 | *B. retrofracta* | Unbalanced apomict | 2C:5C | 1 | 5 | **-** | | **a1** | **1** |
| **21** | JL110 | *B. thompsonii*^††^ | Unbalanced apomict | 2C:5C | 1 | 13 | **s13** | | **a11, a13, a14** | **1, 2, 3, 4** |
| **22** | JL106 | *B. spatifolia* x *stricta* | Unbalanced apomict | 2C:5C | 2 | 14 | **-** | | **a4** | **1, 2** |
| **23** | JL104 | *B. spatifolia* x *stricta* | Unbalanced apomict | 2C:5C | 2 | 25 | **-** | | **a4** | **1** |
| **24** | JL141 | *B. stricta* x *retrofracta* | Balanced apomict | 2C:6C | 2 | 16 | **-** | | **a2** | **1, 2, 3** |
| **25** | JL142 | *B. stricta* x *retrofracta* | Balanced apomict | 2C:6C | 1 | 9 | **-** | | **a2** | **1** |
| **26** | JL143 | *B. stricta* x *retrofracta* | Balanced apomict | 2C:6C | 1 | 11 | **-** | | **a2** | **1** |
| **27** | JL73 | *B. retrofracta* x *stricta*^††^ | Balanced apomict | 2C:6C | 1 | 22 | **s1, s2** | | **a2** | **1, 2, 4** |
| **28** | ES514 | *B. murrayi* x *stricta*^††^ | Balanced apomict | 2C:6C | 1 | 11 | **-** | | **a10** | **1, 2, 3, 4** |
| **29** | ES524 | *B. divaricarpa* | Balanced apomict | 2C:6C | 1 | 11 | **-** | | **a7** | **1, 2** |
| **30** | ES753 | *B. lignifera* | Balanced apomict | 2C:6C | 1 | 10 | **-** | | **a8** | **1** |
| **31** | ES776 | *B. holboellii* | Balanced apomict | 2C:6C | 1 | 7 | **-** | | **a1** | **1, 2, 3** |
| **32** | ES805 | *B. holboellii* | Balanced apomict | 2C:6C | 1 | 12 | **-** | | **a1** | **1, 2** |
| **33** | TS28 | *B. polyantha* | Balanced apomict | 2C:6C | 1 | 11 | **-** | | **a1** | **1, 2** |
| **34** | TS290 | *B. retrofracta* | Balanced apomict | 2C:6C | 1 | 5 | **-** | | **a1** | **1, 2** |
| **35** | TS300 | *B. retrofracta* x *sparsiflora*^††^ | Balanced apomict | 2C:6C | 1 | 7 | **-** | | **a1** | **1, 2, 3, 4** |
| **36** | TS78 | *B. retrofracta* | Balanced apomict | 2C:6C | 1 | 14 | **-** | | **a1** | **1, 2** |
| *Taxon and accession identifiers with ES and TS-prefix taken from Mau et al. (2015) and accession identifiers with JL-prefix from John Lovell (personal communication). Taxa marked with ^††^ were defined in the present study by microsatellite genotyping according to Li et al. (2017). | | | | | | | | | | |
| ^†^ Apomictic male donor accessions with haploid pollen formation = unbalanced apomicts, and with diploid pollen formation = balanced apomicts. | | | | | | | | | | |
| ^‡^ Seed ploidy ratios are inferred from C-values of the embryo and endosperm according to Matzk et al. 2000. | | | | | | | | | | |
| ^§^ Haplotype calls of the *UPGRADE2* gene were classified into sex alleles (s-prefix) and apo alleles (a-prefix). | | | | | | | | | | |
| ^¶^ 1 = *UPG2* haplotype analyses, 2 = *UPG2* promoter haplotype analyses, 3 = RT-qPCR, 4 = Fluorescence in situ hybridization of the *UPG1* and *UPG2* gene amplicons and BAC sequence assemblies (Mau et al. 2013); *UPG2* promoter GUS-eGFP fusion construct transformed into *Arabidopsis thaliana* Col-0. | | | | | | | | | | |

| **Table S2.** Identification of a genetic structure among haplotypes of the *UPGRADE2* gene from sexual, balanced and unbalanced apomictic *Boechera* accessions by hierarchical Bayesian analysis of population structure (BAPS). | | | | | | | | |
| --- | --- | --- | --- | --- | --- | --- | --- | --- |
| **#** | **Sequence identifier** | **Accession** | **Haplotypes** | **Reproductive groups*** | **Haplotype index** | **BAPS cluster level 1^†^** | **BAPS cluster level 2^†^** | **Allele classification^‡^** |
| 1 | ES776_a1_ba | ES776 | a1 | ba | A | 1 | 1 | apo allele |
| 2 | ES805_a1_ba | ES805 | a1 | ba | A | 1 | 1 | apo allele |
| 3 | TS28_a1_ba | TS28 | a1 | ba | A | 1 | 1 | apo allele |
| 4 | TS290_a1_ba | TS290 | a1 | ba | A | 1 | 1 | apo allele |
| 5 | TS300_a1_ba | TS300 | a1 | ba | A | 1 | 1 | apo allele |
| 6 | TS78_a1_ba | TS78 | a1 | ba | A | 1 | 1 | apo allele |
| 7 | JL100_a1_uba | JL100 | a1 | uba | A | 1 | 1 | apo allele |
| 8 | JL99_a1_uba | JL99 | a1 | uba | A | 1 | 1 | apo allele |
| 9 | JL143_a2_ba | JL143 | a2 | ba | B | 2 | 2 | apo allele |
| 10 | JL142_a2_ba | JL142 | a2 | ba | B | 2 | 2 | apo allele |
| 11 | JL141_a2_ba | JL141 | a2 | ba | B | 2 | 2 | apo allele |
| 12 | JL73_a2_ba | JL73 | a2 | ba | B | 2 | 2 | apo allele |
| 13 | JL81_a2_s | JL81 | a2 | s | B | 2 | 2 | apo allele |
| 14 | JL102_a3_uba | JL102 | a3 | uba | C | 3 | 3 | apo allele |
| 15 | JL103_a3_uba | JL103 | a3 | uba | C | 3 | 3 | apo allele |
| 16 | JL104_a4_uba | JL104 | a4 | uba | D | 3 | 4 | apo allele |
| 17 | JL106_a4_uba | JL106 | a4 | uba | D | 3 | 4 | apo allele |
| 18 | JL56_a5_s | JL56 | a5 | s | E | 3 | 3 | apo allele |
| 19 | JL98_a5_uba | JL98 | a5 | uba | E | 3 | 3 | apo allele |
| 20 | JL48_a6_uba | JL48 | a6 | uba | F | 3 | 3 | apo allele |
| 21 | ES758_a6_s | ES758 | a6 | s | F | 3 | 3 | apo allele |
| 22 | JL108_a6_uba | JL108 | a6 | uba | F | 3 | 3 | apo allele |
| 23 | JL107_a6_uba | JL107 | a6 | uba | F | 3 | 3 | apo allele |
| 24 | ES524_a7_ba | ES524 | a7 | ba | G | 3 | 4 | apo allele |
| 25 | ES753_a8_ba | ES753 | a8 | ba | H | 1 | 1 | apo allele |
| 26 | JL6_a9_s | JL6 | a9 | s | I | 3 | 5 | apo allele |
| 27 | ES514_a10_ba | ES514 | a10 | ba | J | 3 | 5 | apo allele |
| 28 | JL110_a11_uba | JL110 | a11 | uba | K | 3 | 5 | apo allele |
| 29 | JL97_a12_uba | JL97 | a12 | uba | L | 3 | 3 | apo allele |
| 30 | JL110_a13_uba | JL110 | a13 | uba | M | 3 | 3 | apo allele |
| 31 | JL110_a14_uba | JL110 | a14 | uba | N | 3 | 5 | apo allele |
| 32 | JL18_a15_s | JL18 | a15 | s | O | 3 | 5 | apo allele |
| 33 | JL48_a16_uba | JL48 | a16 | uba | P | 3 | 3 | apo allele |
| 34 | JL97_a17_uba | JL97 | a17 | uba | Q | 4 | 6 | apo allele |
| 35 | JL48_a18_uba | JL48 | a18 | uba | R | 4 | 6 | apo allele |
| 36 | JL102_a19_uba | JL102 | a19 | uba | S | 4 | 6 | apo allele |
| 37 | ES773_s1_s | ES773 | s1 | s | T | 5 | 7 | sex allele |
| 38 | JL53_s1_s | JL53 | s1 | s | T | 5 | 7 | sex allele |
| 39 | JL73_s1_ba | JL73 | s1 | ba | U | 5 | 7 | sex allele |
| 40 | JL65_s2_s | JL65 | s2 | s | V | 5 | 8 | sex allele |
| 41 | JL73_s2_ba | JL73 | s2 | ba | V | 5 | 8 | sex allele |
| 42 | JL48_s3_uba | JL48 | s3 | uba | W | 5 | 8 | sex allele |
| 43 | JL97_s3_uba | JL97 | s3 | uba | W | 5 | 8 | sex allele |
| 44 | JL82_s4_s | JL82 | s4 | s | X | 5 | 7 | sex allele |
| 45 | JL103_s5_uba | JL103 | s5 | uba | Y | 5 | 7 | sex allele |
| 46 | JL56_s6_s | JL56 | s6 | s | Z | 5 | 8 | sex allele |
| 47 | JL98_s7_uba | JL98 | s7 | uba | AA | 5 | 8 | sex allele |
| 48 | JL12_s8_s | JL12 | s8 | s | AB | 5 | 8 | sex allele |
| 49 | JL12_s9_s | JL12 | s9 | s | AC | 5 | 8 | sex allele |
| 50 | JL45_s10_s | JL45 | s10 | s | AD | 5 | 9 | sex allele |
| 51 | JL56_s10_s | JL56 | s10 | s | AD | 5 | 9 | sex allele |
| 52 | JL45_s11_s | JL45 | s11 | s | AE | 5 | 9 | sex allele |
| 53 | JL45_s12_s | JL45 | s12 | s | AF | 5 | 9 | sex allele |
| 54 | JL110_s13_uba | JL110 | s13 | uba | AG | 5 | 7 | sex allele |
| 55 | ES773_s14_s | ES773 | s14 | s | AH | 5 | 7 | sex allele |
| * Individuals were sorted into groups with a distinct mode of reproduction: s=sexual, ba=balanced apomict and uba=unbalanced apomict. | | | | | | | | |
| † *UPG2* haplotype assignments to hierarchical clusters from first and second level of BAPS clustering | | | | | | | | |
| ^‡^Alleles were classified based on similar SNP distribution and the prevalence of sexual or apomictic *Boechera* accessions in the assigned cluster. | | | | | | | | |

**Table S3.** Percent sequence identities among alleles of the *UPGRADE2* gene in 11 sexual, 12 unbalanced, and 13 balanced apomictic *Boechera* accessions. The allele indices and sequence identifiers are color coded according to the allele clusters identified by hierarchical Bayesian analysis of population structure (BAPS, cf. Fig. 3A).

| **Table S4**. Genetic diversity indices and test of selective neutrality of *UPG2* alleles and *UPG2* promoter sequences for groups of sexual, balanced, and unbalanced apomictic *Boechera* accessions. | | | | | | | | | |
| --- | --- | --- | --- | --- | --- | --- | --- | --- | --- |
| **Location** | **Population** | **N** | **h** | **S** | **Hd (SD)** | **π (SD)** | **θ** | **Neutrality test** | |
|  |  |  |  |  |  |  |  | Tajima's D^†^ | Fu's F* ^†^ |
| *UPG2* gene | sexual | 17 | 14 | 162 | 0.978 (0.027) | 0.015 (0.002) | 0.016 | -0.198 | -0.624 |
|  | unbalanced apomictic | 23 | 14 | 133 | 0.937 (0.033) | 0.013 (0.001) | 0.012 | 0.278 | 0.491 |
|  | balanced apomictic | 15 | 7 | 124 | 0.800 (0.007) | 0.013 (0.002) | 0.013 | 0.231 | 0.599 |
|  | sexual | 8 | 8 | 243 | 1.000 (0.063) | 0.037 (0.005) | 0.034 | 0.535 | 0.504 |
| *UPG2* promoter | unbalanced apomictic | 9 | 5 | 111 | 0.722 (0.159) | 0.010 (0.004) | 0.013 | -1.267 | -1.515 |
|  | balanced apomictic | 10 | 9 | 103 | 0.978 (0.054) | 0.010 (0.004) | 0.012 | -0.628 | 0.012 |
|  | balanced apomictic | 10 | 9 | 103 | 0.978 (0.054) | 0.010 (0.004) | 0.012 | -0.628 | 0.012 |
|  | | | | | | | | | |

| **Table S5.** Genetic heterogeneity statistics and gene flow between *UPG2* alleles in groups of sexual, unbalanced, and balanced apomictic *Boechera* accessions. Genetic differentiation between the reproductive groups was tested by permutation (n=10000) and Chi-square tests. | | | | | | | | | | | | | | | | | | | | | |
| --- | --- | --- | --- | --- | --- | --- | --- | --- | --- | --- | --- | --- | --- | --- | --- | --- | --- | --- | --- | --- | --- |
| **Group 1** | **Group 2** | **Heterogeneity estimates** | | | | | | | | | | **Permutation test^†^** | | ***χ^2^***^††^ | | ***p*-value^#^** | **df** | **Gene flow estimates** | | | |
|  |  | **Kxy** | **G_ST_** | **δ_ST_** | **γ_ST_** | **N_ST_** | **F_ST_** | **Dxy** | **Da** | **Hs** | **Ks** | **Hs *p*-value^#^** | **Ks *p*-value^#^** | |  |  |  | **Nm^‡^** | **Nm^§^** | **Nm^¶^** |  |
| *UPGRADE2* gene | | | | | | | | | | | | | | | | | | | | | |
| balanced apo | unbalanced apo | 44.220 | 0.049 | 0.001 | 0.091 | 0.125 | 0.125 | 0.015 | 0.002 | 0.884 | 38.521 | **0.002**** | **0.009**** | | 29.629 | **0.041*** | 18 | 4.800 | 2.490 | **1.750** |  |
| balanced apo | sexual | 61.757 | 0.049 | 0.004 | 0.215 | 0.314 | 0.312 | 0.021 | 0.006 | 0.895 | 42.628 | **0.002**** | **0.0001***** | | 26.780 | 0.083 | 18 | 4.900 | 0.920 | 0.550 |  |
| unbalanced apo | sexual | 50.772 | 0.005 | 0.002 | 0.125 | 0.183 | 0.182 | 0.017 | 0.003 | 0.954 | 41.085 | 0.190 | **0.006**** | | 31.085 | 0.186 | 25 | 46.370 | 1.750 | **1.110** |  |
| *UPGRADE2* promoter | | | | | | | | | | | | | | | | | | | | | |
| balanced apo | unbalanced apo | 58.833 | 0.075 | 0.005 | 0.358 | 0.473 | 0.473 | 0.019 | 0.009 | 0.859 | 31.050 | **0.014*** | **0.002**** | | 16.994 | 0.150 | 12 | 3.09 | 0.45 | 0.28 |  |
| balanced apo | sexual | 84.938 | -0.006 | 0.005 | 0.194 | 0.222 | 0.221 | 0.031 | 0.007 | 0.987 | 62.009 | 0.815 | **0.009**** | | 13.950 | 0.453 | 14 | -40 | 1.04 | 0.88 |  |
| unbalanced apo | sexual | 90.028 | 0.075 | 0.006 | 0.233 | 0.285 | 0.284 | 0.033 | 0.009 | 0.850 | 62.298 | **0.027*** | **0.002**** | | 17.000 | 0.150 | 12 | 3.07 | 0.82 | 0.63 |  |
| The genomic state for all tested sequences was diploid-autosomic (Fst, γst, Nst = 1 / (1 + 4Nm)). | | | | | | | | | | | | | | | | | | | | | |
| ^†^ Number of permutations = 10000. | | | | | | | | | | | | | | | | | | | | | |
| ^‡^ Gene flow estimates are computed using haplotype data information. If Nm > 1, there is sufficient gene flow to negate the effects of genetic drift, and if Nm > 4, then local populations belong to one panmictic (randomly mating) population (Wright, 1931). | | | | | | | | | | | | | | | | | | | | | |
| ^§^ Gene flow estimates are computed using nucleotide sequence data information (Nei 1982, equation 5). | | | | | | | | | | | | | | | | | | | | | |
| ^¶^ Gene flow estimates are computed using nucleotide sequence data information (Lynch and Crease 1990, Nst uses the Jukes and Cantor (1969) correction). | | | | | | | | | | | | | | | | | | | | | |
| ^#^ p-value: *=p<0.05, **=p<0.01, ***=p<0.001, n.s.= not significant. Values in bold are statistically significant. | | | | | | | | | | | | | | | | | | | | | |
| ^††^ Chi^2^ test for average within population heterogeneity (HS) | | | | | | | | | | | | | | | | | | | | | |

| **Table S6.** Primers used for RT-qPCR, amplification, sequencing and cloning of *UPG2* alleles and putative *UPG2* promoter sites in sexual, balanced and unbalanced apomictic *Boechera* accessions. | | | | | |  |
| --- | --- | --- | --- | --- | --- | --- |
| **Target sequence** | **Experiment** | **Primer name** | **Primer sequence 5`🡪 3`** | **Amplicon size (bp)** | **Amplification efficiency ± SD^‡^** | |
| *UPG2* promoter | Sequencing/amplification | UPG2prom4F  UPG2prom4R | CCCACGATTTTGGAACAATTC  GTTTGATTTCTCTACCTCCACAC | 3473**^*^** | n/a | |
| *UPG2* alleles | Sequencing/amplification | CON234X5-L  CON234X5-R | TCCGACCTAAATCCTACCAAACTGA  TGCTCAATTTTGAACATCTTATTTGC | 3328**^*^** | n/a | |
| *UPG2* alleles | Sequencing/ colony PCR | T3  T7 | ATTAACCCTCACTAAAGGGA  TAATACGACTCACTATAGGG | n/a | n/a | |
| *UPG2* alleles | Sequencing (internal) | 4RBAC_L  4RBAC_R | ATGAACGATGGCGAAGAAGA  TGGATTGCTGTTAAGACCATGT | n/a | n/a | |
| *UPG2* alleles | Sequencing (internal) | CON234X2_L TSP33_R | CTGGAATTGGGTACTTGTATGTCAA  GGTTCGATCCACTGAAGTCCA | n/a | n/a | |
| *UPG2* promoter | Cloning | AttB1-UPG2-F  UPG2-R | GGGGACAAGTTTGTACAAAAAGCAGGCTTCCCACGATTTTGGAACAATTC  CTTGCTCACCATCCGCGGGATATCCTGTGAAAGGGGATCGAGATTAGG | 3231 | n/a | |
| *UPG2* promoter | Cloning | UP-AscI-F  Pst-T35s-R | ATTAGGCGCGCCCACGATTTTGGAACAATTC  TTATCTGCAGTCACTGGATTTTGGTTTTAGG | 5964 | n/a | |
| *UPG2* promoter | Cloning | Napin-PstI-F  DsRed-Kpn-R | TTATCTGCAGCATCGGTGATTGATTCCTT  TTATGGTACCCGATCTAGTAACATAGATG | 2106 | n/a | |
| *UPG2* apoallele | RT-qPCR | UPG2AA-F UPG2ALL-R | CGTTCCTTGATATTTAGTCGGATTTTTGT GGAAAGTCGACGGAAAAGAGCGTTT | 291 | 0.832±0.010 | |
| *UPG2* sexallele | RT-qPCR | UPG2SA-F UPG2ALL-R | TCGTTCCTTGATTTTTTGTCGGAAACT  GGAAAGTCGACGGAAAAGAGCGTTT | 292 | 0.842±0.005 | |
| *UPG2* (allele-unspecific) | RT-qPCR | CON234B4-L  CON234B4-L | TTGCTTTGGTTGAATGCAATAC  AATTACTAAATTTGCACACCACCTG | 177 | 0.839±0.004 | |
| *ACT2* | RT-qPCR | RTActin2T7-F  RTActin2T7-F | GTTCCACCACTGAGCACAATGTTACC  AGTCTTGTTCCAGCCCTCTTTTGTG | 132 | 0.815±0.003 | |
| EF1alpha | RT-qPCR | RTEFα1M13-F  RTEFα1M13-F | CCAAGGGTGAAAGCAAGGAGAGC  CACTGGTGGTTTTGAGGCTGGTATCT | 75 | 0.808±0.002 | |
| ^*^In *Boechera murrayi x stricta* ES514.  ^‡^Efficiencies were calculated with Real Time PCR Miner software v2.0 79. | | | | | |  |

| **Table S7.** Quantitative reverse transcription-PCR analysis of allele-specific expression of the *UPG2* gene in flower bud tissue at meiosis. Distributions are based on the average of four technical replicates from each of four sexual individuals with *UPG2*, three sexual individuals without the gene, three apomictic samples which produce haploid pollen (unbalanced apomicts) and four individuals which produce diploid pollen (balanced apomicts). Different primer combinations were used for the detection of the apo allele (UPG2AA-F and UPG2ALL-R), sex allele (UPG2SA-F and UPG2ALL-R), and both alleles together (CON234B4_L and CON234B4_R; see “Materials and Methods”). The values are means calculated from Ct values of four technical replicates per sample. Relative mRNA expression was normalized against tissue specific tested *Boechera* housekeeping genes *ACTIN2* and *EF1*α*.* | | | | | | | | | | | | | | | | | | | | |
| --- | --- | --- | --- | --- | --- | --- | --- | --- | --- | --- | --- | --- | --- | --- | --- | --- | --- | --- | --- | --- |
| **Accession** | **Individual #** | **Taxon** | **Seed ploidy proportions‡** | | | | | | | | | | | | **N seeds** | **Reproduction** | ***UPGRADE2* alleles†** | **Rel. Expr. Sexallele** | **Rel. Expr. Apoallele** | **Rel. Expr. *UPGRADE2*** |
|  |  |  | **2C:3C** | **2C:4C** | **2C:5C** | **2C:6C** | **2C:7C** | **2C:8C** | **2C:10C** | **3C:6C** | **3C:9C** | **4C:6C** | **5C:6C** | **6C:9C** |  |  |  |  |  |  |
| JL53 | B12-1433 | *B. crandallii* | 1.00 |  |  |  |  |  |  |  |  |  |  |  | 17 | sexual | s1 | 719.45 | 8.11 | 841.30 |
| JL56 | B12-345 | *B. fernaldiana* | 1.00 |  |  |  |  |  |  |  |  |  |  |  | 10 | sexual | s8, s12, a5 | 39.72 | 7.11 | 0.57 |
| ES773 | B12-524 | *B. crandallii* | 0.99 | 0.01 |  |  |  |  |  |  |  |  |  |  | 312* | sexual | s1 | 666.65 | 4.87 | 577.10 |
| ES612 | B13-220 | *B. stricta* | 1.00 |  |  |  |  |  |  |  |  |  |  |  | 312* | sexual | n.d. | 1.30 | 3.43 | 0.29 |
| ES865 | B13-305 | *B. stricta* | 1.00 |  |  |  |  |  |  |  |  |  |  |  | 312* | sexual | n.d. | 18.51 | 3.78 | 0.28 |
| ES913 | B13-363 | *B. polyantha* | 1.00 |  |  |  |  |  |  |  |  |  |  |  | 20 | sexual | n.d. | 1.00 | 1.00 | 1.00 |
| JL103 | B12-1452 | *B. crandallii* | 0.02 |  | 0.92 | 0.01 | 0.05 |  |  | 0.01 |  |  |  |  | 182 | unbalanced apomixis | s5, a3 | 560.19 | 5095.71 | 655.65 |
| JL103 | B12-1453 | *B. crandallii* | 0.02 |  | 0.92 | 0.01 | 0.05 |  |  | 0.01 |  |  |  |  | 182 | unbalanced apomixis | s5, a3 | 1107.16 | 9807.94 | 1828.80 |
| JL110 | B12-1578 | *B. thompsonii* |  |  | 1.00 |  |  |  |  |  |  |  |  |  | 10 | unbalanced apomixis | s16, a11, a13, a14 | 4.83 | 35.61 | 1.84 |
| JL141 | B12-776 | *B. stricta* x *retrofracta* |  |  |  | 1.00 |  |  |  |  |  |  |  |  | 10 | balanced apomixis | a2 | 8.82 | 22.36 | 2.08 |
| ES514 | B13-175 | *B. murrayi* x *stricta* | 0.13 | 0.05 |  | 0.70 |  | 0.03 |  | 0.01 |  | 0.06 | 0.01 |  | 312* | balanced apomixis | a10 | 32.02 | 4990.24 | 298.68 |
| TS300 | B13-170 | *B. retrofracta* x *sparsiflora* |  | 0.04 |  | 0.90 |  |  |  |  | 0.01 | 0.04 |  | 0.01 | 312* | balanced apomixis | a1 | 97.53 | 7714.31 | 574.48 |
| ES776 | B12-2681 | *B. polyantha* |  |  |  | 0.94 |  | 0.05 | 0.01 |  |  |  |  |  | 312* | balanced apomixis | a1 | 86.61 | 8330.03 | 659.85 |
| ‡ Seed ploidy refers to the ratio of the embryo ploidy and the endosperm ploidy. | | | | | | | |  |  |  |  |  |  |  |  |  |  |  |  |  |
| * Values taken from Aliyu et al. 2010. | | |  |  |  |  |  |  |  |  |  |  |  |  |  |  |  |  |  |  |
| † Alleles with s-prefix referred to as 'sex alleles' and the a-prefix refers to 'apo alleles'. | | | | | | | | | | | | | | | | | | | | |

| **Table S8.** Comparison of the genetic structure among alleles of the *UPGRADE2* gene with the *UPG2* promoter in sexual, balanced and unbalanced apomictic *Boechera* accessions by hierarchical Bayesian analysis of population structure (BAPS). | | | | | | | | |  |
| --- | --- | --- | --- | --- | --- | --- | --- | --- | --- |
| **#** | **Sequence identifier** | **Accession** | **Promoter sequence index** | **Reproductive groups*** | **Promoter BAPS cluster level 1^†^** | **Promoter BAPS cluster level 2^†^** | **UPG2 BAPS cluster^‡^** | **UPG2 allele class^§^** | |
| 1 | ES514_a_ba | ES514 | a | ba | 1 | 1 | 3 | apo allele | |
| 2 | JL106_b_uba | JL106 | b | uba | 1 | 1 | 3 | apo allele | |
| 3 | JL18_c_s | JL18 | c | s | 1 | 1 | 3 | sex allele | |
| 4 | JL48_d_uba | JL48 | d | uba | 1 | 2 | mixed | both | |
| 5 | JL97_d_uba | JL97 | d | uba | 1 | 2 | mixed | both | |
| 6 | JL98_d_uba | JL98 | d | uba | 1 | 2 | mixed | both | |
| 7 | JL107_d_uba | JL107 | d | uba | 1 | 2 | 3 | apo allele | |
| 8 | JL108_d_uba | JL108 | d | uba | 1 | 2 | 3 | apo allele | |
| 9 | ES776_e_ba | ES776 | e | ba | 2 | 4 | mixed | apo allele | |
| 10 | JL103_f_uba | JL103 | f | uba | 1 | 2 | 2 | both | |
| 11 | JL141_g_ba | JL141 | g | ba | 1 | 1 | 3 | apo allele | |
| 12 | ES524_h_ba | ES524 | h | ba | 2 | 4 | 5 | apo allele | |
| 13 | JL45_i_s | JL45 | i | s | 1 | 3 | 1 | sex allele | |
| 14 | ES773_j_s | ES773 | j | s | 2 | 5 | 5 | sex allele | |
| 15 | ES805_j_ba | ES805 | j | ba | 2 | 5 | 1 | apo allele | |
| 16 | JL99_k_uba | JL99 | k | uba | 2 | 4 | 1 | apo allele | |
| 17 | TS78_l_ba | TS78 | l | ba | 2 | 4 | 1 | apo allele | |
| 18 | JL65_m_s | JL65 | m | s | 2 | 4 | 5 | sex allele | |
| 19 | TS28_n_ba | TS28 | n | ba | 2 | 5 | 1 | apo allele | |
| 20 | JL110_o_uba | JL110 | o | uba | 1 | 3 | 1 | both | |
| 21 | TS290_p_ba | TS290 | p | ba | 2 | 4 | mixed | apo allele | |
| 22 | JL73_q_ba | JL73 | q | ba | 2 | 6 | 1 | both | |
| 23 | TS300_r_ba | TS300 | r | ba | 2 | 6 | 3 | apo allele | |
| 24 | JL6_s_s | JL6 | s | s | 1 | 3 | mixed | apo allele | |
| 25 | ES758_t_s | ES758 | t | s | 3 | 7 | 3 | apo allele | |
| 26 | JL12_u_s | JL12 | u | s | 3 | 7 | 5 | sex allele | |
| 27 | JL53_v_s | JL53 | v | s | 3 | 7 | 5 | both | |
| * Individuals were sorted into groups with a distinct mode of reproduction: s=sexual, ba=balanced apomict and uba=unbalanced apomict. | | | | | | | | |  |
| ^†^ *UPG2* promoter allele assignments to hierarchical clusters from first and second level of BAPS clustering. | | | | | | | | |  |
| ^‡^Overlay of the five BAPS haplotype cluster from the *UPG2* gene onto the *UPG2* promoter allele cluster. 'Mixed' = *UPG2* promoter haplotype is assigned to multiple *UPG2* gene BAPS clusters | | | | | | | | |  |
| ^§^ Assignments of *UPG2* sex and apo allele classification to *UPG2* promoter allele clusters. 'Both' = Accession with sex and apo alleles of the *UPG2* gene aligns on a single UPG2 promoter allele. | | | | | | | | |  |

| **Table S9.** Locus by locus AMOVA of the *UPGRADE2* gene and promoter sequence between sexual, balanced and unbalanced apomictic *Boechera* accessions. | | | | | | | |
| --- | --- | --- | --- | --- | --- | --- | --- |
| **Locus** | **Source of variation** | **Sum of squares** | **Variance components** | **% variation** | **Fst** | **P-value*** |  |
| *UPG2* gene | Among reproductive groups | 505.067 | 5.926 | 20.91 | 0.209 | 0.0000 |  |
|  | Within reproductive groups | 2277.514 | 22.419 | 79.09 |  |  |  |
|  | Total | 2782.582 | 28.345 |  |  |  |  |
| *UPG2* promoter | Among reproductive groups | 579.677 | 13.157 | 32.11 | 0.321 | 0.0000 |  |
|  | Within reproductive groups | 1323.975 | 27.820 | 67.89 |  |  |  |
|  | Total | 1903.652 | 40.977 |  |  |  |  |
| *Significance tests were run with 10100 permutations | | | | | | | |

| **Supplemental table 10.** Transcription factor (TF) binding site prediction for the *UPGRADE2* promoter region between -3143nt and -1nt relative to the *UPGRADE2* transcription start site. The binding site prediction function of the PlantRegMap web browser (http://plantregmap.gao-lab.org, p-value cutoff ≤1e-4) was used to identify all potential interactions between known TFs from a sexual *Boechera stricta* genome (v1.2, DOE-JGI, http://phytozome.jgi.doe.gov/) and the three query consensus sequences of the *UPGRADE2* promoter region in sexual, balanced and unbalanced apomicts. | | | | | | | | | | |
| --- | --- | --- | --- | --- | --- | --- | --- | --- | --- | --- |
| **TF family** | **TFBS in *UPG2* promoter*** | | | |  | **TF gene count** | | **Over- (>) or underrepresentation (<) of TF genes binding on UPG2pro** | **Probability statistics** | |
|  | **Sexual** | **Balanced apo** | **Unbalanced apo** | **Total** |  | ***UPG2pro*** | ***B. stricta* JGI v1.2** |  | ***p*-value** | **Chi2-Total** |
| AP2 | 5 | 4 | 10 | 19 |  | 2 | 20 | < | 0.758 | 0.43 |
| ARF | 17 | 15 | 15 | 47 |  | 4 | 24 | > | 1 | 0.003 |
| B3 | 11 | 9 | 12 | 32 |  | 4 | 67 | **<** | **0.036** | 4.08 |
| BBR-BPC | 14 | 10 | 29 | 53 |  | 3 | 13 | > | 0.479 | 0.31 |
| bHLH | 5 | 7 | 5 | 17 |  | 7 | 170 | **<** | **0.000** | 14.70 |
| bZIP | 4 | 7 | 4 | 15 |  | 6 | 86 | **<** | **0.031** | 4.22 |
| C2H2 | 35 | 30 | 44 | 109 |  | 14 | 109 | < | 0.500 | 0.67 |
| C3H | 1 | 1 | 1 | 3 |  | 1 | 55 | **<** | **0.003** | 6.89 |
| CAMTA | 3 | 1 | 1 | 5 |  | 2 | 6 | > | 0.309 | 0.82 |
| CPP | 4 | 4 | 6 | 14 |  | 4 | 8 | > | 0.073 | 3.76 |
| Dof | 31 | 35 | 25 | 91 |  | 17 | 37 | **>** | **0.001** | 13.86 |
| E2F/DP | 3 | 3 | 3 | 9 |  | 2 | 10 | > | 0.679 | 0.07 |
| EIL | 2 | 2 | 2 | 6 |  | 1 | 6 | > | 1 | 0.001 |
| ERF | 53 | 53 | 51 | 157 |  | 38 | 126 | **>** | **0.001** | 11.66 |
| FAR1 | 1 | 0 | 1 | 2 |  | 1 | 22 | < | 0.237 | 1.76 |
| G2-like | 12 | 12 | 13 | 37 |  | 3 | 55 | **<** | **0.053** | 3.71 |
| GATA | 6 | 7 | 3 | 16 |  | 4 | 35 | < | 0.644 | 0.44 |
| GRAS | 8 | 7 | 12 | 27 |  | 1 | 39 | **<** | **0.035** | 4.36 |
| GRF | 1 | 1 | 1 | 3 |  | 1 | 8 | < | 1 | 0.06 |
| HD-ZIP | 16 | 16 | 12 | 44 |  | 11 | 52 | > | 0.457 | 0.65 |
| LBD | 4 | 7 | 2 | 13 |  | 7 | 49 | < | 1 | 0.10 |
| MIKC_MADS | 18 | 18 | 22 | 58 |  | 11 | 48 | > | 0.337 | 1.09 |
| MYB | 80 | 80 | 64 | 224 |  | 36 | 147 | **>** | **0.024** | 5.02 |
| MYB_related | 12 | 12 | 12 | 36 |  | 11 | 77 | < | 0.874 | 0.15 |
| NAC | 16 | 17 | 22 | 55 |  | 23 | 128 | > | 0.624 | 0.21 |
| NF-YB | 1 | 1 | 1 | 3 |  | 1 | 18 | < | 0.501 | 1.19 |
| Nin-like | 2 | 2 | 2 | 6 |  | 1 | 18 | < | 0.501 | 1.19 |
| RAV | 4 | 4 | 4 | 12 |  | 4 | 6 | > | 0.039 | 5.66 |
| TCP | 4 | 4 | 4 | 12 |  | 4 | 25 | < | 1 | 0.0005 |
| Trihelix | 20 | 20 | 21 | 61 |  | 8 | 34 | > | 0.364 | 0.91 |
| WOX | 2 | 2 | 1 | 5 |  | 2 | 16 | < | 1 | 0.12 |
| WRKY | 22 | 22 | 22 | 66 |  | 22 | 80 | **>** | **0.027** | 4.95 |
| YABBY | 1 | 1 | 1 | 3 |  | 1 | 6 | > | 1 | 0.001 |
| ZF-HD | 2 | 4 | 3 | 9 |  | 5 | 18 | > | 0.356 | 1.17 |
| *A consensus sequence of the *UPGRADE2* promoter region (-3143nt to -1nt relative to TSS in *B. murrayi* x *stricta* ES514) from each 8 sexual, 11 balanced and 8 unbalanced apomictic *Boechera* accessions was used as query. The transcription factor genes and families were in *Boechera stricta* v1.2 (DOE-JGI, http://phytozome.jgi.doe.gov/). | | | | | | | | | | |
| † Two-sided Fishers exact test | | | | | | | | | | |
